# Serial Gene Expression Profiling of Neural Stem Cells Shows Transcriptome Switch by Long-Term Physioxia from Metabolic Adaption to Cell Signaling Profile

**DOI:** 10.1155/2022/6718640

**Published:** 2022-11-12

**Authors:** Lena Braunschweig, Jennifer Lanto, Anne K. Meyer, Franz Markert, Alexander Storch

**Affiliations:** ^1^Division of Neurodegenerative Diseases, Department of Neurology, Technische Universität Dresden, 01307 Dresden, Germany; ^2^Center for Regenerative Therapies Dresden (CRTD), Technische Universität Dresden, 01307 Dresden, Germany; ^3^Department of Neurology, University of Rostock, 18147 Rostock, Germany; ^4^German Centre for Neurodegenerative Diseases (DZNE) Rostock/Greifswald, 18147 Rostock, Germany

## Abstract

Oxygen is an essential factor in the cellular microenvironment with pivotal effects on neural development with a particular sensitivity of midbrain neural stem cells (NSCs) to high atmospheric oxygen tension. However, most experiments are still performed at atmospheric O_2_ levels (21%, normoxia), whereas mammalian brain tissue is physiologically exposed to substantially lower O_2_ tensions around 3% (physioxia). We here performed serial Affymetrix gene array analyses to detect expression changes in mouse fetal NSCs from both midbrain and cortical tissues when kept at physioxia compared to normoxia. We identified more than 400 O_2_-regulated genes involved in cellular metabolism, cell proliferation/differentiation, and various signaling pathways. NSCs from both regions showed a low number but high conformity of regulated genes (9 genes in midbrain vs. 34 in cortical NSCs; 8 concordant expression changes) after short-term physioxia (2 days) with *metabolic processes* and *cellular processes* being the most prominent GO categories pointing to cellular adaption to lower oxygen levels. Gene expression profiles changed dramatically after long-term physioxia (13 days) with a higher number of regulated genes and more diverse expression patterns when comparing the two NSC types (338 genes in midbrain vs. 121 in cortical NSCs; 75 concordant changes). Most prominently, we observed a reduction of hits in *metabolic processes* but an increase in *biological regulation* and *signaling* pointing to a switch towards signaling processes and stem cell maintenance. Our data may serve as a basis for identifying potential signaling pathways that maintain stem cell characteristics in cortical versus midbrain physioxic stem cell niches.

## 1. Introduction

Multipotent tissue-specific neural stem cells (NSCs) with their capacity for self-renewal and multilineage differentiation potentially guarantee increasing cell numbers and diversity during fetal brain development as well as neurogenesis in adult neurogenic brain regions [1, 2]. There is a high interest in NSCs and their derivatives as promising tools for stem cell therapies, tissue engineering, pharmacological testing, and modelling of the development of the brain and peripheral nervous system tissue [3–8]. Studies of the microenvironmental demands of these NSCs within the neurogenic niche are thus crucial to maintain their survival, proliferation, and differentiation potential *in vitro* and *in vivo* [9–15]. Oxygen tension is one key factor of the neurogenic niche regulating cellular functions in stem cells, and lowered oxygen tension of around 3% is beneficial for stem cell proliferation, survival, and maintenance in various NSC types both *in vitro* and *in vivo* [16–21]. Indeed, the physiological oxygen tension within the mammalian brain ranges from 1% to 5% O_2_ (known as physioxia [22, 23]) and thus differs remarkably from the atmospheric oxygen condition routinely used in *in vitro* culture [24–26]. Moreover, sensitivity to oxygen tension seems to vary between NSCs from different fetal brain regions with NSCs isolated from the midbrain showing a selective vulnerability to oxygen as compared to cortical NSCs (cNSCs) [18, 27, 28]: reduced O_2_ is vital to *in vitro* cultures of fetal midbrain-derived NSCs (mNSCs) along with proliferation, survival, and potential to dopaminergic differentiation [16, 18, 19, 28, 29].

The mechanisms of oxygen sensitivity of NSCs including the differences between the various NSC types remain however in part enigmatic. Moreover, there are some inconsistencies about the underlying factors which may be caused by differences not only of the tissue of origin of the NSCs but also in the oxygen conditions with levels close to anoxia (<1% O_2_) and/or in the cultivation periods in altered oxygen tensions [30–32]. In physioxia, hypoxia-inducible factors (Hifs) are the main regulators of O_2_ homoeostasis through their target genes such as *vascular endothelial growth factor* (*Vegf*) or *phosphoglycerate kinase 1* (*Pgk1*) inducing the hypoxic response including increased angiogenesis or altered glucose metabolism [29, 33–35]. Consistently, neural-specific Hif-1*α* conditional knock-out models showed that expression of Hif-1*α* in neural cells is essential for the normal development of the brain [36, 37]. Moreover, in our previous study, we demonstrated a selective vulnerability of mNSCs against Hif-1*α* conditional knock-out leading to impaired proliferation, survival, and dopaminergic differentiation solely of mNSCs [28]. Another signaling pathway involved on oxygen-mediated NSC behavior, which is independent of Hif-1*α*, is the Wnt/*β*-catenin pathway positively stimulating proliferation of physioxic cells by affecting cell cycle regulation [27].

In the present study, we focus on whole transcriptome analyses using gene chip microarrays of fetal mesencephalic and cortical NSCs cultured in normoxia (21% atmospheric oxygen tension) or physioxia (3% oxygen tension) to provide a framework for future studies on the molecular mechanisms of oxygen adaptation of various types of fetal NSC involved in stem cell proliferation, survival, and maintenance.

## 2. Materials and Methods

### 2.1. Neural Stem Cell Isolation, Culture, and Treatments

For the present whole microarray analyses, we used the biosamples published previously by Braunschweig and colleagues in 2015 [27]. In brief, wild-type C57Bl/6J mice were purchased from Charles River, Sulzfeld, Germany, and animal procedures were approved by the Animal Rights Committee. Embryos at day 14 were removed by Caesarean section, and mesencephalic and cortical brain regions were dissected. After removal of the meninges, the tissue samples were trypsinized (2.5 ng/ml, Sigma-Aldrich, Seelze, Germany) for 10 min at room temperature, incubated in DNase (40 *μ*g/ml, Sigma-Aldrich, Seelze, Germany) for 10 min at 37°C, and subsequently triturated to achieve a single-cell suspension. For monolayer cultures, cells were plated onto poly-L-ornithine/fibronectin-precoated culture dishes and maintained in an expansion medium composed of DMEM (high glucose) supplemented with 32% F12, 2% B27, and 1% penicillin/streptomycin (all from Life Technologies, Darmstadt, Germany) and 20 ng/ml EGF and FGF2 (Sigma-Aldrich, Hamburg, Germany). Medium change was performed three times per week. For the hypoxic culture, cells were maintained in a gas mixture composed of 92% N_2_, 5% CO_2_, and 3% O_2_. To preserve constant O_2_ levels, the medium was preequilibrated by exposure to the gas mixture for at least 24 hours. NSCs were used for experiments directly after isolation without cell culture passaging.

### 2.2. Isolation of RNA

Isolation of RNA was carried out using the RNeasy® Mini Kit (Qiagen, Hilden, Germany) according to manufacturer's instruction. To avoid adaption of the cells to the atmospheric O_2_ level, the initiate steps were done quickly (within 6 min). RNA was eluted in RNase-free water by centrifugation, and RNA concentration was determined with the Quant-iT™ RNA Assay Kit (Life Technologies Corporation, Darmstadt, Germany) according to the manufacturer's instruction. For later analysis, RNA was stored at -80°C.

### 2.3. Quantitative RT-PCR

Quantitative RT-PCR was performed using the SYBR Green PCR Kit (Qiagen, Hilden, Germany) and the Stratagene Mx3000P thermocycler with the following program: 95°C for 15 min followed by 40 cycles of 94°C for 15 sec, 55°C for 30 sec, and 72°C for 30 sec. *Hydroxymethylbilane synthase* (*Hmbs*) was used as housekeeping genes for normalization. Initial studies on housekeeping gene expression in mNSCs over the cultivation time at the different oxygen levels revealed stable expression of *Hmbs* but not other housekeeping genes such as *Glycerinaldehyd-3-phosphat-dehydrogenase* (*Gapdh*) or *Tyrosine 3-monooxygenase/tryptophan 5-monooxygenase activation protein zeta* (*Ywhaz*; Supplementary Figure S1). The following primers were used: *Hes5* F, 5′-GCTCGCTAATCGCCTCCA-3′; *Hes5* R, 5′-GGTCCCGACGCATCTTCT-3′; *Hif3a* F, 5′-CACTGCTCAGGACATATGAG-3′; *Hif3a* R, 5′-TCCAAAGCGTGGATGTATTC-3′; *Hmbs* F, 5′-TGTATGCTGTGGGTCAGGGAG-3′; *Hmbs* R, 5′-CTCCTTCCAGGTGCCTCAGA-3′; *Igfbp4* F, 5′-GGTAGATCTTTGCTGTGGGAA-3′; *Igfbp4* R, 5′-CCTCAGACACACACAATCCAA-3′; *Ldha* F, 5′-GGGTATCTCTGTGTAGCCCTGA-3′; *Ldha* R, 5′-AGAGGCAGGTGGATGTCTGT-3′; *Pdk1* F, 5′-GGTTGGGAACCACTCTTTCA-3′; *Pdk1* R, 5′-GCTTTGGTTACGTGGCATTT-3′; *Sfrp5* F, 5′-GATCTGTGCCCAGTGTGAGA-3′; *Sfrp5* R, 5′-TTCAGCTGCCCCATAGAAA-3′; *Slc2a1* F, 5′-CGACCCTCTTCTTTCATCTCCT-3′; *Slc2a1* R, 5′-CAGGTCTCGGGTCACATCG-3′; *Slc2a3* F, 5′-GAACATTGGCTGGATACCTCTA-3′; *Slc2a3* R, 5′-ACAGTCACGGCGAACACC-3′; *Vegf* F, 5′-GCTACTGCCGTCCGATTG-3′; and *Vegf* R, 5′-CTCCAGGGCTTCATCGTTAC-3′ (all from Eurofins Genomics, Ebersberg, Germany).

### 2.4. Microarray Gene Chip Analysis

For whole microarray analyses, we used the microarray data from Braunschweig and coworkers [27]. As described in our previous article, mesencephalic and cortical NSCs were maintained in the expansion medium for 2 or 13 days under strict O_2_ conditions using preequilibrated medium. After isolation of RNA from three independent cultures per condition and cell type, the concentration was measured using a Nanodrop spectrometer (Agilent Technologies, Santa Clara, CA, USA), and RNA quality was approved by an Agilent 2100 Bioanalyzer (all samples had a RNA integrity number > 9.0). Subsequent hybridization to whole mouse genome microarray gene expression chips (Gene ST 1.0 Arrays, Affymetrix, Santa Clara, CA, USA) was performed following the manufacturer's protocol. Microarray chips were then immediately scanned using the Hewlett-Packard Gene Array Scanner G2500A (Agilent Technologies, Santa Clara, CA, USA). Raw data were processed with the Exon Array Computational Tool (ExACT; Affymetrix) for background correction and normalization, and statistical data analysis was performed in the Agilent GeneSpring GX11 software. Statistical significance was computed using the unpaired Student's *t*-test (*P* < 0.05) with Benjamini-Hochberg as the multiple testing correction.

### 2.5. Gene Ontology Classification Analysis

The genes, which were up- or downregulated in the microarray analyses with a fold change of ≥2, were analysed in a Gene Ontology analysis via the website “geneontology.org,” which is using the PANTHER 17.0 Classification System software (http://pantherdb.org/). Of note, setting the fold change threshold to 1.5 led to very similar results. In this process, the regulated genes are classified into the different GO domains: biological process, molecular function, and cellular component, through manual and electronic annotations generated by a computer algorithm based on sequence similarities. The resulting genes are then allocated into different subgroups according to their biological context and occurrence. Based on the number of genes, the percentage of each subgroup in the entire GO domain can be determined. The resulting hits were composed of the diverse biological functions of a gene. For the analysis of the pathways, the PANTHER and Reactome databases were used. Reactome is a manually classified and peer-reviewed pathway database, but it also works in conjunction with PANTHER to identify orthologues of human proteins annotated in Reactome. Fisher's exact tests and Bonferroni correction were used in the PANTHER software to calculate fold enrichment and *P* values [38, 39].

## 3. Results

### 3.1. Differential Gene Regulation by Physioxia in Midbrain versus Cortical NSCs

**Figure 1:**
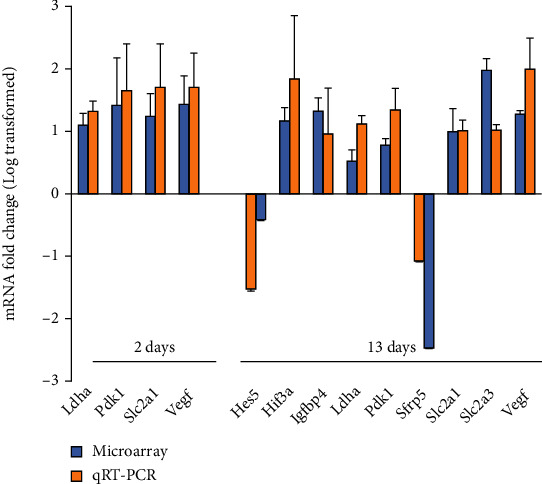
Comparison of gene expression levels measured with microarray gene chip assay and qRT-PCR showing good correlations between both measurements in mNSC. Log2-transformed fold changes in mRNA levels (3% O_2_/21% O_2_) and standard error of mean (*n* = 3) were plotted for both array and qRT-PCR.

To identify possible stem cell factors and molecular pathways differentially involved in the O_2_ adaptation of fetal NSCs derived from midbrain and cortical tissues, Affymetrix microarray gene chip arrays were performed with NSCs cultured in physioxic 3% O_2_ or in normoxic 21% O_2_ for a short-term (2 days) or a long-term period (13 days). These whole-transcript assays provide a complete expression profile of 28,853 coding transcripts in the mouse genome. To avoid overinterpretation of the data, the threshold for significance was set at a twofold or greater change in the expression level for all genes analysed (for complete lists of up- and downregulated genes, refer to Supplementary Tables S1–S6). Several of these genes were chosen to confirm fidelity of the microarray data by quantitative RT-PCR. Consistent results in the mode of regulation could be found in all cases proving reliability of the gene chip assays (Figure 1).

For both time points and also for both tissues, we detected more genes that were upregulated than downregulated in the physioxic compared to the normoxic condition (Table 1). None of the 28,853 investigated genes were downregulated in cells which were kept in physioxia for a 2-day period, whereas significant upregulation of expression could be observed for 9 genes in mesencephalic NSC and 34 genes in cortical NSC when kept at low O_2_ levels for 2 days. Upon long-term culturing for 13 days, the number of significantly upregulated genes was 226 in midbrain and 96 in cortical NSCs, while 112 genes and 25 genes were downregulated, respectively.

**Figure 2:**
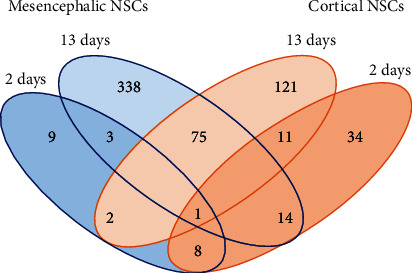
Comparison of differentially expressed genes in short- versus long-term physioxia of mesencephalic and cortical NSC cultures. The 4-way Venn diagram shows total numbers of O_2_-regulated genes when comparing mesencephalic with cortical NSCs and short- (2 days) versus long-term O_2_ stimulation (13 days).

Regarding short-term physioxia, the transcriptomes of midbrain and cortical NSCs showed a high number of identical genes compared with the total number of regulated genes, indicating strict concordance in gene expression. Here, 8 out of 9 more than twofold upregulated genes in the midbrain were also significantly upregulated in the cortex after short-term physioxia (Figure 2). However, long-term physioxia changed the gene expression profiles dramatically. There was an increase in affected genes in both cultures, with the number of matched genes being only 75 out of 338 genes in midbrain cells that were also affected in cortical cells. Interestingly, only 2 out of 9 genes that were induced by short-term physioxia in the midbrain were also significantly upregulated in long-term physioxic midbrain cells. These comparisons revealed that especially long-term physioxia influenced the gene expression pattern.

### 3.2. PANTHER Classification according to Biological Process of the Genes Regulated by Oxygen Levels in Midbrain versus Cortical NSCs

**Figure 3:**
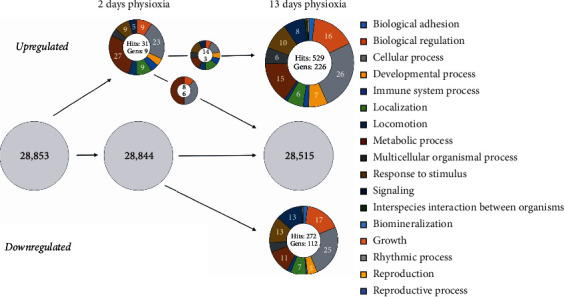
Global comparison of PANTHER gene categories “biological process” in midbrain NSC cultures. Displayed are percentages of hits from oxygen-regulated genes to specific categories according to the PANTHER domain “biological process.” The PANTHER classification includes the total up-/downregulated genes (lower number in the inner circle) with at least a twofold difference for short- and long-term cultures. The grey circles indicate all analysed genes without a change of expression due to O_2_ treatment. After 2 days of culture (2 d), no gene was downregulated in hypoxic cells, but 9 genes from different categories were upregulated, which formed a total of 31 hits. 3 genes and 14 hits of them were also upregulated in hypoxic prolonged cultures, while the other 6 genes did not undergo any O_2_ change in prolonged cultures. In total, 226 genes were upregulated and formed 529 hits, and 112 genes with 272 hits were downregulated after 13 days of hypoxic culture (13 d). The hits result from the multiple biological functions of a gene. Since a gene can also have multiple functions, one gene also often results in multiple hits. Also shown are the percentages of individual hits in the various biological functions (categories) to the total hits of the regulated genes.

**Figure 4:**
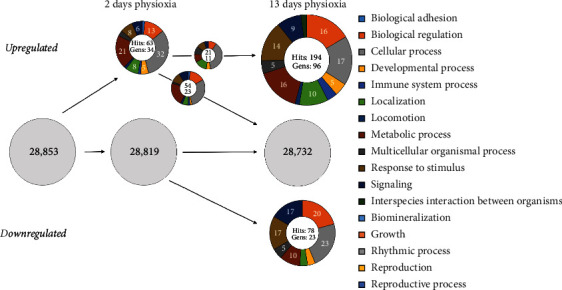
Global comparison of PANTHER gene categories “biological process” in cortical NSC cultures. Displayed are percentages of hits from oxygen-regulated genes to specific categories according to the PANTHER domain “biological process.” The PANTHER classification includes the total up-/downregulated genes (lower number in the inner circle) with at least a twofold difference for short- and long-term cultures. The grey circles indicate all analysed genes without a change of expression due to O_2_ treatment. After 2 days of culture (2 d), no gene was downregulated in hypoxic cells, but 9 genes from different categories were upregulated, which formed a total of 31 hits. 3 genes and 14 hits of them were also upregulated in hypoxic prolonged cultures, while the other 6 genes did not undergo any O_2_ change in prolonged cultures. In total, 226 genes were upregulated and formed 529 hits, and 112 genes with 272 hits were downregulated after 13 days of hypoxic culture (13 d). The hits result from the multiple biological functions of a gene. Since a gene can also have multiple functions, one gene also often results in multiple hits. Also shown are the percentages of individual hits in different biological functions (categories) to the total hits of the regulated genes.

We then categorized all O_2_-regulated genes using the PANTHER classification system (http://pantherdb.org/) according to the biological process (PANTHER GO-Slim Biological Process). Since individual genes have multiple functions and are listed in multiple groups, this total number is referred to as hits. The different classes are represented by the percentage of summed hits in the respective biological process category normalized to total hits. The individual hits of the categories represent the individual genes of the categories. The global comparison between short- and long-term cultures showed that their gene expression profiles differed over a wide range from the 28,853 genes analysed (Figures 3 and 4). In general, we observed a rise in the number of total categories as well as the sum of all hits, which indicates an increase in the varieties of biological processes.

In the case of short-term physioxia midbrain cultures, the analysed genes can be functionally classified into the two large categories: *metabolic processes* (27% of all hits, 6 hits) and *cellular processes* (23% of all hits, 6 hits) which are represented by genes such as *Pdk1* (pyruvate dehydrogenase kinase 1) or *Tpi1* (triose phosphate isomerase 1; Figure 3). The number of hits increased from 31 to 801 (up- and downregulated) under long-term physioxia as compared to the normoxia condition leading to much bigger diversity of biological processes (increase in categories from 10 to 15). Despite various additional categories (*biomineralization*, *locomotion*, and *growth*), *cellular processes* (23%, 5 hits to 26%, 135 hits) and *biological regulation* (9%, 2 hits to 16%, 84 hits) even increased in the long-term hypoxic condition. On the other side, total hits in this category are increased, while the proportion of *metabolic processes* decreased from 27% (6 hits) to 15% (80 hits) at long term in midbrain NSC cultures.

Similar results were observed in short-term physioxic cortical NSCs, where 34 out of the 28,853 analysed genes are upregulated (Figure 4). The 63 attributed hits can be classified once more into *cellular processes* (32%, 20 hits) and *metabolic processes* (21%, 13 hits) representing the two largest categories as shown in short-term physioxic midbrain cultures. In long-term cortical cultures, although not reaching the level of hits from midbrain results, the hits increased to 274 (up- and downregulated). Here, the expression patterns differed from midbrain cultures since both *cellular processes* (32%, 20 hits to 17%, 34 hits) and *metabolic processes* (21%, 20 hits to 16%, 34 hits) are reduced when compared to overall hits, and the distribution of categories is even more diverse than that in the midbrain. Next, there was a slight increase of hits from 13% (8 hits) in short-term to 16% (32 hits) in long-term physioxic cortical cells in the category *biological regulation* and also an increase of the *response to stimulus* category (8%, 5 hits to 14%, 28 hits).

Regarding long-term downregulated genes and their hits, both cultures showed very similar patterns to their hits from upregulated genes: a large percentage of genes belonged to the biological process categories *biological regulation* (midbrain: 17%, 46 hits; cortex: 20%, 16 hits), *cellular processes* (midbrain: 25%, 67 hits; cortex: 23%, 18 hits), and *response to stimulus* (midbrain: 13%, 34 hits; cortex: 17%, 13 hits). Although the distribution of hits still showed a high regulation in the category *signaling* (midbrain: 13%, 34 hits; cortex: 17%, 13 hits), the category was particularly prominent in cortical NSCs. The *signaling* category is represented by genes like *Hey2* (hairy/enhancer-of-split related with YRPW motif protein 2) and *Dll1* (delta-like protein 1) both involved in *Notch signaling* and thus playing a crucial role in the development of the nervous system [40, 41].

In summary, more genes in midbrain and cortex NSCs were regulated by a prolonged physioxic environment as compared with short-term physioxia. Most prominently, there was a reduction of hits in *metabolic processes* but an increase in *biological regulation* and *signaling* when compared to overall hits. Despite many differentially regulated genes, the differences in categories and their distribution between midbrain and cortical cultures were small.

### 3.3. GO Classification of the Genes Regulated by Oxygen Levels in Midbrain versus Cortical NSCs

We then analysed the regulated genes for their classification into the GO domains of biological processes, molecular functions, and cellular components using GO analysis (“geneontology.org”), which also uses the PANTHER database. Overall, detailed analyses of biological processes, molecular functions, and cellular components at short-term physioxia revealed primarily a link to *glycolysis*, *pyruvate*, and *general carbohydrate metabolism* regardless of the tissue origin (Supplementary Table S7). Genes specifically responsible for the cellular response to low oxygen conditions were also upregulated in both cultures. In this context, the upregulated genes also showed effects on peptidyl proline 4-dioxygenase (or prolyl 4-hydroxylase), which is generally required for the hydroxylation of prolyl residues in proteins such as collagen and, in turn, for the stabilization of hypoxia-inducible factor-1 (HIF-1) [42–44]. Its activity is controlled by several genes, such as *Egln3* (prolyl hydroxylase) and *P4ha1* (prolyl 4-hydroxylase subunit alpha-1), which were also upregulated in both cultures after short-term physioxia.

The analysis of long-term cultures showed that the fold enrichment of the individual biological processes, cellular functions, and components was rather low compared to the results in short-term physioxia (Supplementary Tables S8 and S9). On closer inspection, the cellular process *protein hydroxylation* seems to be very present in the upregulated genes under long-term physioxia in a tissue-independent manner (fold enrichment of at least 20.1; Supplementary Table S8). Both NSC types reflected genes associated with the *binding of collagen* as the molecular function, which also played a role in the cellular components of the basement membrane (“*collagen-containing extracellular matrix*”). In contrast, only midbrain NSC cultures had regulated gene functions in angiogenesis and neuron generation after prolonged exposure to physioxia. Interestingly, in the midbrain, genes that positively influence *Notch signaling* and *neurogenesis* appear to be downregulated (Supplementary Table S9).

### 3.4. GO Cellular Pathway Analyses of the Genes Regulated by Oxygen Levels in Midbrain versus Cortical NSCs

We then categorized the regulated genes in relation to cellular metabolic pathways using GO (Gene Ontology) analysis via geneontology.org using the two types of software and databases PANTHER and Reactome. Analysis of the 9 upregulated genes in short-term midbrain NSC cultures revealed that two of these genes with a fold enrichment greater than 100 were associated with a glycolysis pathway, and 3 genes were associated with a pyruvate pathway, which is required, for example, for the functioning of the citrate acid cycle (Table 2). Cortical cultures also revealed pathway affiliation with *glycolysis*. In contrast to midbrain cultures, cortical cells showed no affiliation in *pyruvate metabolism* but an additional effect regarding *collagen formation*.

In long-term physioxic NSC cultures, it was generally noticed that the fold enrichment of the cellular pathways was comparatively low compared to the results in short-term physioxia (Tables 3 and 4). The pathway affiliations of midbrain cells showed relations to *Posttranslational protein phosphorylation* and *Regulation of Insulin-like Growth Factor (IGF) transport and uptake by Insulin-like Growth Factor Binding Proteins (IGFBPs)*. Both pathways could not be found in GO pathway analyses from affected genes in cortical NSCs but showed a link to *amino acid transport across the plasma membrane* with a fold enrichment of 28.63.

Pathway analysis of the downregulated genes in long-term physioxia revealed an association with *Notch signaling* in midbrain (fold enrichment: 18.7) as well as cortical NSCs (fold enrichment: 62.8; Table 4). Once more, there are differences between both cultures since affected genes of midbrain cells are associated with *Wnt signaling*: *Frzb* (secreted frizzled-related protein 3), *Sfrp5* (secreted frizzled-related protein 5), and *Nkd1* (protein naked cuticle homolog 1). In contrast, affected genes of cortical NSCs are associated with *transmission across chemical synapses* demonstrating once more a tissue-dependent reaction to long-term physioxia.

### 3.5. Differential Regulation of Differentiation Marker Genes by Physioxia in Midbrain NSC

**Figure 5:**
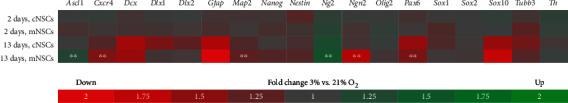
Comparison of differentiation-related genes in short- versus long-term physioxia of mesencephalic and cortical NSC cultures. The heat map shows slight changes in fate decisions solely in long-term physioxic mNSC (after 13 days). Fold changes of differentiation-related genes are displayed using colour coding (red: downregulated; green: upregulated). ^∗∗^*P* < 0.01, *t*-test with Benjamini-Hochberg procedure for multiple testing correction.

To further characterize the cNSC and mNSC cultured in 3% O_2_ and used for microarray analysis, we displayed a set of specific genes corresponding to neuronal differentiation (Figure 5). The analysis of these markers revealed that lowered oxygen levels did not affect differentiation markers in short-term cultures. On the other hand, there are changes in long-term cultures, which reaches significance solely in mNSCs: two of these markers—*Gfap* and *Map2*—were downregulated, which is comparable with our previous immunohistochemical data [27]. *Ngn2* as a prominent marker for differentiation into various neuronal subtypes was also downregulated [45]. Intriguingly, our data suggested that lowered oxygen levels promote a slight transition of mNSC towards oligodendrocyte precursor cells (OPCs) indicated by significant upregulation of the OPC proliferation regulator *Ascl1* and the OPC-specific marker *Ng2*, while the mature OPC marker *Olig2* is not affected while the radial glia marker *Pax6* is downregulated [46, 47].

## 4. Discussion

Global comparison of gene expression profiles of short-term (2 days) and long-term (13 days) NSC cultures derived from fetal midbrain or cortical tissue showed that initial adaptation to O_2_ deficiency is distinct from maintenance of the response to physioxic culture conditions as suggested in previous reports [48, 49].

In the short-term (2 days) cultures of midbrain and cortical NSCs, very few genes exhibited a twofold or greater change in gene expression upon O_2_ alteration, relative to the 28,853 genes examined. There was a high concordance of individual genes in both NSC types. The O_2_-regulated genes mainly involve glycolysis and contribute to the current knowledge that cells reduce glucose production in the presence of decreasing O_2_ through a three-step switch from oxidative to glycolytic metabolism to maintain ATP levels [50–52]. The first step involves activated transcription of glucose transporters and glycolytic enzymes to increase glycolytic flux from glucose to pyruvate. Second, the expression of pyruvate dehydrogenase kinase 1 (Pdk1) is induced to prevent flux through the tricarboxylic acid cycle and decrease the production of reactive oxygen species. And third, transcription of lactate dehydrogenase A (*Ldha*) is induced to activate the conversion of pyruvate to lactate. Therefore, the switch is known to be regulated via hypoxia-inducible factors (HIFs) which are key regulators for low oxygen conditions [53–55]. Consequently, whole-transcriptome analyses showed that relevant genes such as Pdk1 and the glucose transporter *Slc2a1* (solute carrier family 2, member 1) were upregulated, leading to altered biological processes such as pyruvate and glucose metabolism, but also the cellular response to physioxia from gene ontology analysis. These results, consistent with the literature, suggest a largely tissue-independent regulation of cells to short-term hypoxia/physioxia.

The direct response to hypoxia/physioxia of both NSC cultures is also evident in the upregulation of the molecular function peptidyl proline 4-dioxygenase activity involving the *P4ha1* and *Egln3* (*Phd3*) genes. *P4ha1*, only regulated in short-term hypoxia, is known to induce HIF-1 signaling in breast cancer cells through hypoxia [44]. *Phd3* encodes an intracellular prolyl hydroxylase that, distinct from *Phd1* or *Phd2*, primarily hydroxylates the alfa subunit of hypoxia-inducing factor 2 (HIF-2a) acting as a putative feedback control or fast acting regulator in the case of reoxygenation [56–61]. In such normoxic conditions, HIF proteins are continuously hydroxylated by HIF-prolyl hydroxylases (PHD1-3) and degraded by the vHL protein (Von Hippel-Lindau protein). Since these hydroxylation reactions require oxygen, the degradation of HIFs decreases at lower oxygen tension, allowing them to transcribe target genes as transcription factors [62, 63]. Of note, the *Phd3* mRNA level is elevated independent of tissue or physioxia duration, although there are dramatic changes in long-term physioxia gene expression. Thus, our findings likely contribute to the knowledge that it acts as a fast regulator for the case of reoxygenation [64].

While the cellular response to short-term physioxia seems to be related to immediate metabolic changes for cell survival, long-term physioxic (13 days) cultures showed a fundamentally different scenario: from the perspective of chronic physioxia, it may become more important for the cells to prepare for a prolonged absence of O_2_ than attempting to further improve supply [26, 32, 65]. Consecutively, the concordance of genes between short-term and long-term physioxia within the same culture (mesencephalon or cortex) was less than 33%, and hypoxia response genes such as *Vegf*, glucose transporter, or *Pdk1* are less or absent in NSCs cultured in long-term physioxia. This results in a switch from affected metabolic pathways in short-term physioxic cultures to signaling pathways such as Notch signaling or Wnt signaling, which is consistent with human studies showing that the response to short-term hypoxia/physioxia differs substantially from adaptation to long-term hypoxia/physioxia [48, 49]. Intriguingly, the affected signaling pathways Notch and Wnt (only in midbrain NSCs) are key players within embryonic brain development *in vivo* [66, 67]. Associated genes such as *Dll3*, *Hes5*, *Frzb*, or *Cdh8* are all known to be important for adequate brain development and support the relevance of so-called hypoxic niches where neural stem and progenitor cells reside *in vivo* [68, 69]. *Dll1*, *Dll3*, or *Hes5* as part of Notch signaling and downregulated in long-term physioxic midbrain NSC is known to be essential for neurogenesis and is predominantly described for maintenance of stem cell state and blocking of differentiation [70–77]. In long-term physioxic cortical NSCs, similar results were found regarding Notch signaling by downregulation of Dll1 or Hey2 [78, 79]. Although most of these studies are only relevant for the cortical situation, they are conflicting with enhanced proliferation under hypoxia from previous reports [27]. This may be explained by the oscillation of Notch genes such as *Dll1* or *Hes1* whose sustained expression would lead to inhibition of proliferation [80, 81].

In long-term physioxic NSC cultures from the midbrain, Wnt signaling is also affected through downregulated genes such as *Celsr1*, *Sfrp5*, *Frzb*, or *Cdh8*. Here, for example, *Celsr1*, *Frzb* (also known as *Sfrp3*) and Sfrp5 (or its homologs *Sfrp1/2*) functional studies revealed that their inhibition is linked to enhanced stem cell maintenance (Boucherie [82–85]. Vice versa, we could detect upregulated proliferation-enhancing genes of the Wnt pathway like *Ccnd2* known to be expressed during midbrain development [86–88]. We thus conclude that known proliferative effects on midbrain NSC through physioxia might be mediated through Wnt pathways and differ compared to cortical cells. These results are consistent with our previous functional results [16, 27]. Additionally, our analysis of differentiation markers revealed a reduction of *Map2* expression in physioxia, which is consistent with our previous results [27]. This effect is solely detectable in mNSCs, which—in contrast to cNSCs—show nearly no spontaneous differentiation towards Map2^+^ cells after cultivation in physioxia [27]. Interestingly, we also observed a slight shift of OPC marker expression towards immature OPCs.

Besides Wnt, we could detect other specific pathwayssuch as *Posttranslational protein phosphorylation* and *Regulation of Insulin-like Growth Factor (IGF) transport and uptake by Insulin-like Growth Factor Binding Proteins (IGFBPs)* for midbrain NSCs. As an example, *Igfbp4* is normally expressed during rodent brain development but starts to significantly decrease with E14.5 *in vivo* [89]. At this stage, brain vascularization—beginning with its first sprouting at roughly E9—is established, and oxygen can be easily delivered, which may act as a signaling factor during development [36, 90, 91]. Additionally, the upregulated genes *Igf2* and *Igfbp3* are linked to human brain development while *Igfbp3* in particular is able to regulate cell growth at least [92, 93]. Of note, despite GO not revealing this pathway in cortical NSCs, its counterpart *Igfbp4* is also regulated in cortical cells.

Moreno and colleagues found calcineurin-NFATc4 signaling in hypoxic mouse NSCs as an additional signaling pathway acting as a major regulator of self-renewal and proliferation [32]. We have no indication of an importance of this transcription factor pathway in our NSC cultures (fold change of Nfatc4 in mNSC after 48 h: 1.00; mNSC, 13 d: 1.01; cNSC, 48 h: 1.09; cNSC, 13 d: 1.00). This discrepancy might be due to differences in the experimental paradigm: we here used primarily freshly isolated NSC at 3% for physioxia, while Moreno and coworkers cultured their NSCs at 5% oxygen tension over several cell culture passages. While it is well known that passaging can provoke changes regarding the cellular properties, Horie and coworkers showed—at least for NSC from the ganglionic eminence—that there is a critical shift of NSC behavior at around 4% O_2_ which might explain the mentioned differences [94, 95]. Moreover, Moreno and colleagues performed a gene set enrichment analysis (GSEA) using transcription factor target (TFT) and KEGG gene sets. Unfortunately, both gene sets are currently not available for the murine situation in the GSEA software limiting a direct comparison of our data.

Cortical NSCs remarkably show affected pathways regarding *amino acid transport across the plasma membrane* and *transmission across chemical synapses*. Our previous results show that there are only sporadic differentiated cells in long-term physioxic cortical cells [27], so we assume that the regulation of chemical synapses corresponds to signaling regulating stem cell properties rather than its classical role in neuronal functionality. In particular, gamma-aminobutyric acid (GABA) signaling is reported to play a distinct role during brain—particularly cortical—development [96–99]. In agreement with the literature [32, 99], we detected the expression of various GABA receptor subunits with downregulation in long-term physioxia of *Gabbr1* (subunit 1 of the GABA_B_ receptor) and *Gabrg2* (subunit *γ*2 of the GABA_A_ receptor) in cortical but only *Gabbr2* (subunit 2 of the GABA_B_ receptor) in midbrain NSCs. In contrast, *Gabra2* (subunit *α*2 of the GABA_A_ receptor) was upregulated in cortical NSC in physioxic conditions. Interestingly, both GABA receptor subtypes have been reported to regulate NSC proliferation [96, 97]. However, there are conflicting results by showing that GABA could also express inhibitory effects on proliferation of cortical progenitors [98]. The effects of GABA on NSC proliferation, maintenance, and differentiation might thus depend on the exact developmental stage of the NSCs and their microenvironment [97, 99].

## 5. Conclusion

This work focuses on changes in gene expression in midbrain and cortical NSCs that occur under low O_2_ culture conditions reflecting physiological oxygen tension and which are beneficial expansion conditions for fetal NSCs as compared to atmospheric O_2_. The study shows specific effects of oxygen on NSCs during short-term cultivation mostly affecting metabolic processes which dramatically shifts to signaling processes and stem cell maintenance during long-term cultivation. Strikingly, even though short-term effects are very similar in both NSC types, midbrain and cortical NSCs react differently during long-term cultivation in their physioxic environment underpinning the discrepancies in their oxygen response [16, 18, 19, 28]. These results reveal the extent of molecular mechanisms during O_2_ modulation and help to identify pathways that control mammalian stem cell behavior in hypoxic/physioxic stem cell niches.

## Figures and Tables

**Table 1:** Number of genes up- or downregulated more than twofolds in NSCs in physioxia.

	Midbrain NSCs (2 days in culture)	Cortical NSCs (2 days in culture)	Midbrain NSCs (13 days in culture)	Cortical NSCs (13 days in culture)
Upregulated in physioxia (3% O_2_)	9	34	226	96
Downregulated in physioxia (3% O_2_)	0	0	112	25

Displayed are the numbers of regulated genes for the various culture conditions and origins of NSCs. For complete gene lists, refer to Supplementary Tables S1–S6.

**Table 2:** Affected pathways from upregulated genes in midbrain and cortex NSCs cultivated for 2 days in physioxia.

	Midbrain NSCs				Cortical NSCs		
	Number of genes	Fold enrichment	P value		Number of genes	Fold enrichment	P value
PANTHER pathways *Mus musculus* (reference)				PANTHER pathways *Mus musculus* (reference)			
Glycolysis	2	>100	8.10E-03	—			
Reactome pathways *Mus musculu*s (reference)				Reactome pathways *Mus musculu*s (reference)			
Pyruvate metabolism	3	>100	3.41E-04	Glycolysis	4	43.11	4.54E-03
				(i) Glucose metabolism	4	32.34	1.35E-02
				(ii) metabolism of carbohydrates	6	14.59	5.45E-03
				Collagen formation	4	29.40	1.94E-02

**Table 3:** Affected pathways from upregulated genes in midbrain and cortex NSCs cultivated for 13 days in physioxia.

	Midbrain NSCs				Cortical NSCs		
	Number of genes	Fold enrichment	P value		Number of genes	Fold enrichment	P value
PANTHER pathways *Mus musculus* (reference)				PANTHER pathways *Mus musculus* (reference)			
—				Amino acid transport across the plasma membrane	4	28.63	2.86E-02
Reactome pathways *Mus musculu*s (reference)				Reactome pathways *Mus musculu*s (reference)			
Regulation of IGF transport and uptake by IGF binding proteins	10	8.25	1.25E-03	—			
Post-translational protein phosphorylation	9	7.82	6.76E-03				

**Table 4:** Affected pathways from downregulated genes in midbrain and cortex NSCs cultivated for 13 days in physioxia.

	Midbrain NSCs				Cortical NSCs		
	Number of genes	Fold enrichment	P value		Number of genes	Fold enrichment	P value
PANTHER pathways *Mus musculus* (reference)				PANTHER pathways *Mus musculus* (reference)			
Notch signaling pathway	4	18.70	1.35E-02	Notch signaling pathway	3	62.82	2.78E-03
Wnt signaling pathway	8	4.99	3.79E-02	—			
Reactome pathways *Mus musculu*s (reference)				Reactome pathways *Mus musculu*s (reference)			
—				Transmission across chemical synapses	5	20.55	6.78E-03

## Data Availability

All data including microarray data can be obtained by contacting the corresponding author without any restrictions.

## Supplementary Materials

Supplementary Materials Supplementary Figure S1: comparison of cycle threshold (CT) values of various housekeeping genes as measured by qRT-PCR in dependency on cultivation times and oxygen levels in mesencephalic NSCs. Supplementary Table S1: upregulated genes in cortical NSCs cultivated for 2 days in physioxia compared to normoxia. Supplementary Table S2: upregulated genes in cortical NSCs cultivated for 13 days in physioxia compared to normoxia. Supplementary Table S3: downregulated genes in cortical NSCs cultivated for 13 days in physioxia compared to normoxia. Supplementary Table S4: upregulated genes in midbrain NSCs cultivated for 2 days in physioxia compared to normoxia. Supplementary Table S5: upregulated genes in midbrain NSCs cultivated for 13 days in physioxia compared to normoxia. Supplementary Table S6: downregulated genes in midbrain NSCs cultivated for 13 days in physioxia compared to normoxia. Supplementary Table S7: affected biological processes, molecular functions, and components from upregulated genes in midbrain and cortical NSCs cultivated for 2 days in physioxia. Supplementary Table S8: affected biological processes, molecular functions, and components from upregulated genes in midbrain and cortical NSCs cultivated for 13 days in physioxia. Supplementary Table S9: affected biological processes, molecular functions, and components from downregulated genes in midbrain and cortical NSCs cultivated for 13 days in physioxia.
