# Supplementary material for: Serial Gene Expression Profiling of Neural Stem Cells Shows Transcriptome Switch by Long-Term Physioxia from Metabolic Adaption to Cell Signaling Profile
Source: Stem Cells Int. 2022 Nov 12;2022:6718640. doi: 10.1155/2022/6718640 (PMC9675612; doi:10.1155/2022/6718640)
Supplement: Supplementary Materials — Supplementary Figure S1: comparison of cycle threshold (CT) values of various housekeeping genes as measured by qRT-PCR in dependency on cultivation times and oxygen levels in mesencephalic NSCs. Supplementary Table S1: upregulated genes in cortical NSCs cultivated for 2 days in physioxia compared to normoxia. Supplementary Table S2: upregulated genes in cortical NSCs cultivated for 13 days in physioxia compared to normoxia. Supplementary Table S3: downregulated genes in cortical NSCs cultivated for 13 days in physioxia compared to normoxia. Supplementary Table S4: upregulated genes in midbrain NSCs cultivated for 2 days in physioxia compared to normoxia. Supplementary Table S5: upregulated genes in midbrain NSCs cultivated for 13 days in physioxia compared to normoxia. Supplementary Table S6: downregulated genes in midbrain NSCs cultivated for 13 days in physioxia compared to normoxia. Supplementary Table S7: affected biological processes, molecular functions, and components from upregulated genes in midbrain and cortical NSCs cultivated for 2 days in physioxia. Supplementary Table S8: affected biological processes, molecular functions, and components from upregulated genes in midbrain and cortical NSCs cultivated for 13 days in physioxia. Supplementary Table S9: affected biological processes, molecular functions, and components from downregulated genes in midbrain and cortical NSCs cultivated for 13 days in physioxia. [file 6718640.f1.doc]

**Serial gene expression profiling of neural stem cells shows transcriptome switch by long-term physioxia from metabolic to stem cell signaling profile**

Lena Braunschweig, Jennifer Lanto, Anne K. Meyer, Franz Markert, and Alexander Storch

Supplementary Material

**Supplementary Figure:**

- **Supplementary Figure S1: Comparison of cycle threshold (CT)-values of various housekeeping genes as measured by qRT-PCR in dependency on cultivation times and oxygen levels in mesencephalic NSCs.**

**Supplementary Tables:**

- **Supplementary Table S1: Upregulated genes in cortical NSCs cultivated for 2 days in physioxia compared to normoxia.**
- **Supplementary Table S2: Upregulated genes in cortical NSCs cultivated for 13 days in physioxia compared to normoxia.**
- **Supplementary Table S3: Downregulated genes in cortical NSCs cultivated for 13 days in physioxia compared to normoxia.**
- **Supplementary Table S4: Upregulated genes in midbrain NSCs cultivated for 2 days in physioxia compared to normoxia.**
- **Supplementary Table S5: Upregulated genes in midbrain NSCs cultivated for 13 days in physioxia compared to normoxia.**
- **Supplementary Table S6: Downregulated genes in midbrain NSCs cultivated for 13 days in physioxia compared to normoxia.**
- **Supplementary Table S7: Affected biological processes, molecular functions and components from upregulated genes in midbrain and cortical NSCs cultivated for 2 days in physioxia.**
- **Supplementary Table S8: Affected biological processes, molecular functions and components from upregulated genes in midbrain and cortical NSCs cultivated for 13 days in physioxia.**
- **Supplementary Table S9: Affected biological processes, molecular functions and components from downregulated genes in midbrain and cortical NSCs cultivated for 13 days in physioxia.**


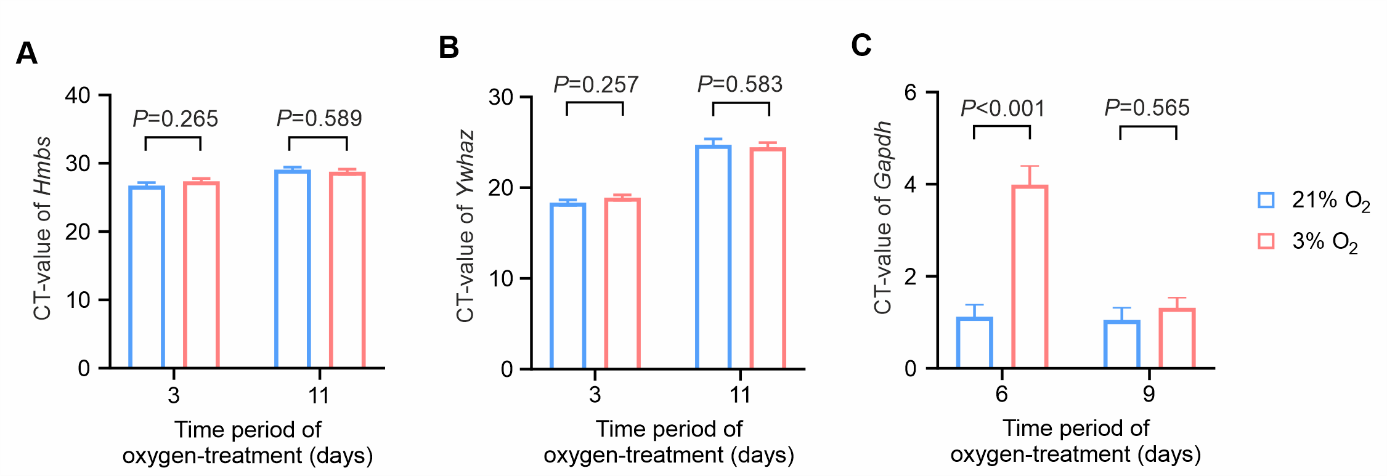


**Supplementary Figure S1: Comparison of cycle threshold (CT)-values of various housekeeping genes as measured by qRT-PCR in dependency on cultivation times and oxygen levels in mesencephalic NSCs**. Initial amounts of RNA were quantified via Nano-Drop 1000. *Hmbs, Hydroxymethylbilane synthase; Gapdh, Glycerinaldehyd-3-phosphat-dehydrogenase; Ywhaz, Tyrosine 3-monooxygenase/tryptophan 5-monooxygenase activation protein zeta*. *P*-values for the comparison of oxygen tensions are from two-way ANOVA and *post-hoc t*-tests with atmospheric oxygen tension and cultivation time period as fixed factors (n=4-5; *P-*values ≥ 0.05 are considered non-significant).

**Supplementary Table S1: Upregulated genes in cortical NSCs cultivated for 2 days in physioxia as compared to normoxia.**

| **Cortical NSCs (2 days) – Upregulated in 3% O_2_ (34 genes)** | | | |
| --- | --- | --- | --- |
| **No.** | **Gene symbol** | **Gene name** | **Fold change** |
| 1 | Slc16a3 | solute carrier family 16 (monocarboxylic acid transporters). member 3 | 19.749 |
| 2 | Bnip3 | BCL2/adenovirus E1B interacting protein 1. NIP3 | 7.605 |
| 3 | Pdk1  (Q8BFP9) | pyruvate dehydrogenase kinase. isoenzyme 1 | 6.705 |
| 4 | Egln3 | EGL nine homolog 3 (C. elegans) | 5.367 |
| 5 | Slc2a1 | solute carrier family 2 (facilitated glucose transporter). member 1 | 3.732 |
| 6 | Adm | adrenomedullin | 3.653 |
| 7 | Vegfa | vascular endothelial growth factor A | 3.491 |
| 8 | Pfkl | phosphofructokinase. liver. B-type | 3.461 |
| 9 | Slc2a3 | solute carrier family 2 (facilitated glucose transporter). member 3 | 3.308 |
| 10 | Stc2 | stanniocalcin 2 | 3.226 |
| 11 | Prelid2 | PRELI domain containing 2 | 3.038 |
| 12 | P4ha1 | procollagen-proline. 2-oxoglutarate 4-dioxygenase (proline 4-hydroxylase). alpha 1 polypeptide | 2.856 |
| 13 | Loxl2 | lysyl oxidase-like 2 | 2.848 |
| 14 | Tpi1 | triosephosphate isomerase 1 | 2.752 |
| 15 | Ndufa4l2 | NADH dehydrogenase (ubiquinone) 1 alpha subcomplex. 4-like 2 | 2.679 |
| 16 | Car9 | carbonic anhydrase 9 | 2.663 |
| 17 | Ache  (P21836) | acetylcholinesterase | 2.613 |
| 18 | Pgm2|Pgm1  (NM_028132) | phosphoglucomutase 2 | phosphoglucomutase 1 | 2.556 |
| 19 | Pfkp | phosphofructokinase. platelet | 2.553 |
| 20 | Higd1a | HIG1 domain family.member 1A|similar to hypoxia induced gene 1 | 2.458 |
| 21 | Pfkfb3 | 6-phosphofructo-2-kinase/fructose-2.6-biphosphatase 3 | 2.339 |
| 22 | A2m  (Q6GQT1) | alpha-2-macroglobulin | 2.305 |
| 23 | Ddit4 | DNA-damage-inducible transcript 4 | 2.304 |
| 24 | Ero1l | ERO1-like (S. cerevisiae) | 2.190 |
| 25 | Txnip | thioredoxin interacting protein | 2.147 |
| 26 | P4ha2 | procollagen-proline. 2-oxoglutarate 4-dioxygenase (proline 4-hydroxylase). alpha II polypeptide | 2.127 |
| 27 | Dsc1 | desmocollin 1 | 2.103 |
| 28 | Jhdm1d | jumonji C domain-containing histone demethylase 1 homolog D (S. cerevisiae) | 2.099 |
| 29 | Kbtbd11 | kelch repeat and BTB (POZ) domain containing 11 | 2.092 |
| 30 | Igfbp5 | insulin-like growth factor binding protein 5 | 2.058 |
| 31 | Jmjd1a | jumonji domain containing 1A | 2.055 |
| 32 | Grhpr | glyoxylate reductase/hydroxypyruvate reductase | 2.039 |
| 33 | Smtnl2 | smoothelin-like 2 | 2.032 |
| 34 | Col12a1 | collagen. type XII. alpha 1 | 2.022 |

**Supplementary Table S2: Upregulated genes in cortical NSCs cultivated for 13 days in physioxia compared to normoxia.**

| **Cortical NSCs (13 days) - Upregulated in 3% O_2_ (96 Genes)** | | | |
| --- | --- | --- | --- |
|  | **Gene symbol** | **Gene name** | **Fold change** |
| 1 | Clic5 | chloride intracellular channel 5 | 5.034 |
| 2 | Slc7a3 | solute carrier family 7 (cationic amino acid transporter. y+ system). member 3 | 4.970 |
| 3 | Slc7a11 | solute carrier family 7 (cationic amino acid transporter. y+ system). member 11 | 4.836 |
| 4 | Slc38a4 | solute carrier family 38. member 4 | 4.764 |
| 5 | Cd93 | CD93 antigen | 4.442 |
| 6 | Nptx1 | neuronal pentraxin 1 | 4.028 |
| 7 | Dhh | desert hedgehog | 3.869 |
| 8 | Raet1b | retinoic acid early transcript beta | 3.792 |
| 9 | H60b | histocompatibility 60b | 3.792 |
| 10 | Igfbp4 | insulin-like growth factor binding protein 4 | 3.470 |
| 11 | Gpc3 | glypican 3 | 3.384 |
| 12 | Mamdc2 | MAM domain containing 2 | 3.359 |
| 13 | Ctsh | cathepsin H | 3.313 |
| 14 | Fgf1 | fibroblast growth factor 1 | 3.311 |
| 15 | Col12a1 | collagen. type XII. alpha 1 | 3.285 |
| 16 | Ier3 | immediate early response 3 | 3.284 |
| 17 | Arhgap29 | Rho GTPase activating protein 29 | 3.240 |
| 18 | Rpp25 | ribonuclease P 25 subunit (human) | 3.147 |
| 19 | Lama4 | laminin. alpha 4 | 3.142 |
| 20 | Unc93b1 | unc-93 homolog B1 (C. elegans) | 3.127 |
| 21 | Chl1 | cell adhesion molecule with homology to L1CAM | 3.126 |
| 22 | Ddr2 | discoidin domain receptor family. member 2 | 3.110 |
| 23 | Phospho1 | phosphatase. orphan 1 | 3.106 |
| 24 | Ifitm2 | interferon induced transmembrane protein 2 | 3.060 |
| 25 | Cox7a1 | cytochrome c oxidase. subunit VIIa 1 | 2.962 |
| 26 | Ndufa4l2 | NADH dehydrogenase (ubiquinone) 1 alpha subcomplex. 4-like 2 | 2.874 |
| 27 | Trf (Q921I1) | transferrin | 2.873 |
| 28 | Leprel1 (P3h2) | leprecan-like 1 | 2.858 |
| 29 | Aldh1l2 | aldehyde dehydrogenase 1 family. member L2 | 2.849 |
| 30 | Cda | cytidine deaminase | 2.791 |
| 31 | Tmem56 | transmembrane protein 56 | 2.764 |
| 32 | Pck2 | phosphoenolpyruvate carboxykinase 2 (mitochondrial) | 2.745 |
| 33 | Klf10 | Kruppel-like factor 10 | 2.728 |
| 34 | Lrrc32 | leucine rich repeat containing 32 | 2.722 |
| 35 | Stc1 | stanniocalcin 1 | 2.716 |
| 36 | Slc22a3 | solute carrier family 22 (organic cation transporter). member 3 | 2.704 |
| 37 | H2-Ab1 | histocompatibility 2. class II antigen A. beta 1 | 2.688 |
| 38 | Arc (Q9WV31) | activity regulated cytoskeletal-associated protein | 2.641 |
| 39 | Layn | layilin | 2.592 |
| 40 | Prelid2 | PRELI domain containing 2 | 2.566 |
| 41 | Car13 | carbonic anhydrase 13 | 2.548 |
| 42 | Kcnn4 | potassium intermediate/small conductance calcium-activated channel. subfamily N. member 4 | 2.542 |
| 43 | Cacng3 | calcium channel. voltage-dependent. gamma subunit 3 | 2.541 |
| 44 | Ifitm3 | interferon induced transmembrane protein 3 | 2.517 |
| 45 | Tm4sf1 | transmembrane 4 superfamily member 1 | 2.504 |
| 46 | Ero1l | ERO1-like (S. cerevisiae) | 2.484 |
| 47 | Pcbd1 | pterin 4 alpha carbinolamine dehydratase/dimerization cofactor of hepatocyte nuclear factor 1 alpha (TCF1) 1 | 2.483 |
| 48 | Stc2 | stanniocalcin 2 | 2.477 |
| 49 | Rara | retinoic acid receptor. alpha | 2.473 |
| 50 | Pir | pirin | 2.464 |
| 51 | Gdf15 | growth differentiation factor 15 | 2.437 |
| 52 | Asns | asparagine synthetase | 2.437 |
| 53 | Ldb2 | LIM domain binding 2 | 2.408 |
| 54 | Gypc | glycophorin C | 2.404 |
| 55 | Trib1 | tribbles homolog 1 (Drosophila) | 2.373 |
| 56 | Hmox1 | heme oxygenase (decycling) 1 | 2.370 |
| 57 | Fkbp10 | FK506 binding protein 10 | 2.357 |
| 58 | Kdelr3 | KDEL (Lys-Asp-Glu-Leu) endoplasmic reticulum protein retention receptor 3 | 2.356 |
| 59 | Chac1 | ChaC. cation transport regulator-like 1 (E. coli) | 2.351 |
| 60 | Cyb5r2 | cytochrome b5 reductase 2 | 2.349 |
| 61 | Kcnj16 | potassium inwardly-rectifying channel. subfamily J. member 16 | 2.340 |
| 62 | Ramp1 | receptor (calcitonin) activity modifying protein 1 | 2.336 |
| 63 | Unc5b | unc-5 homolog B (C. elegans) | 2.323 |
| 64 | Gabra2 | gamma-aminobutyric acid (GABA-A) receptor subunit alpha 2 | 2.314 |
| 65 | Nupr1 | nuclear protein 1 | 2.306 |
| 66 | Cd302 | CD302 antigen | 2.298 |
| 67 | Sec24d | SEC24 related gene family. member D (S. cerevisiae) | 2.286 |
| 68 | Ucp2 | uncoupling protein 2 (mitochondrial. proton carrier) | 2.271 |
| 69 | Stat6 | signal transducer and activator of transcription 6 | 2.253 |
| 70 | Aox1 | aldehyde oxidase 1 | 2.249 |
| 71 | Myadm | myeloid-associated differentiation marker | 2.247 |
| 72 | Mthfd1l | methylenetetrahydrofolate dehydrogenase (NADP+ dependent) 1-like | 2.239 |
| 73 | Cd97 (Adgre5) | CD97 antigen | 2.217 |
| 74 | Adm | adrenomedullin | 2.213 |
| 75 | Slc2a3 | solute carrier family 2 (facilitated glucose transporter). member 3 | 2.213 |
| 76 | Slc16a3 | solute carrier family 16 (monocarboxylic acid transporters). member 3 | 2.212 |
| 77 | Ctsz | cathepsin Z | 2.210 |
| 78 | Itih5 | inter-alpha (globulin) inhibitor H5 | 2.197 |
| 79 | Pfkp | phosphofructokinase. platelet | 2.169 |
| 80 | Mthfd2 | methylenetetrahydrofolate dehydrogenase (NAD+ dependent) | 2.167 |
| 81 | Ccdc80 | coiled-coil domain containing 80 | 2.154 |
| 82 | Ache (P21836) | acetylcholinesterase | 2.140 |
| 83 | Egln3 | EGL nine homolog 3 (C. elegans) | 2.116 |
| 84 | Cpne7 | copine VII | 2.103 |
| 85 | Slc7a1 | solute carrier family 7 (cationic amino acid transporter. y+ system). member 1 | 2.098 |
| 86 | Hectd2 | HECT domain containing 2 | 2.098 |
| 87 | Creb3l1 | cAMP responsive element binding protein 3-like 1 | 2.095 |
| 88 | Plod2 | procollagen lysine. 2-oxoglutarate 5-dioxygenase 2 | 2.089 |
| 89 | Plagl1 | pleiomorphic adenoma gene-like 1 | 2.073 |
| 90 | Pdzd2 | PDZ domain containing 2 | 2.051 |
| 91 | Bst2 | bone marrow stromal cell antigen 2 | 2.051 |
| 92 | Kcnc4 | potassium voltage gated channel. Shaw-related subfamily. member 4 | 2.036 |
| 93 | Plp2 | proteolipid protein 2 | 2.035 |
| 94 | Atf5 | activating transcription factor 5 | 2.016 |
| 95 | Ripk3 | receptor-interacting serine-threonine kinase 3 | 2.005 |
| 96 | Crot | carnitine O-octanoyltransferase | 2.003 |

**Supplementary Table S3: Downregulated genes in cortical NSCs cultivated for 13 days in physioxia compared to normoxia.**

| **Cortex 13d - Downregulated in 3% O_2_ (25 genes)** | | | |
| --- | --- | --- | --- |
|  | **Gene symbol** | **Gene name** | **Fold change** |
| 1 | Abca1 | ATP-binding cassette. sub-family A (ABC1). member 1 | 3.294 |
| 2 | Hey2 | hairy/enhancer-of-split related with YRPW motif 2 | 3.100 |
| 3 | Pde9a | phosphodiesterase 9A | 3.039 |
| 4 | Igsf11 | immunoglobulin superfamily. member 11 | 3.024 |
| 5 | Abat | 4-aminobutyrate aminotransferase | 2.754 |
| 6 | Plcd4 | phospholipase C. delta 4 | 2.660 |
| 7 | Naaa | Nacylethanolamine acid amidase | 2.487 |
| 8 | Adcy8 | adenylate cyclase 8 | 2.473 |
| 9 | Stmn2 | stathmin-like 2 | 2.439 |
| 10 | Gabbr1 | gamma-aminobutyric acid (GABA-B) receptor 1 | 2.338 |
| 11 | Mc5r | melanocortin 5 receptor | 2.302 |
| 12 | Sncaip | synuclein. alpha interacting protein (synphilin) | 2.296 |
| 13 | Pde1b | phosphodiesterase 1B. Ca2+-calmodulin dependent | 2.293 |
| 14 | Btg2 | B-cell translocation gene 2. anti-proliferative | 2.279 |
| 15 | Dll1 | delta-like 1 (Drosophila) | 2.242 |
| 16 | Pak3 | p21 (CDKN1A)-activated kinase 3 | 2.225 |
| 17 | Mfng | MFNG O-fucosylpeptide 3-beta-N-acetylglucosaminyltransferase | 2.194 |
| 18 | Prkcq | protein kinase C. theta | 2.189 |
| 19 | Lix1 | limb expression 1 homolog (chicken) | 2.166 |
| 20 | Cadps | Ca2+-dependent secretion activator | 2.149 |
| 21 | Mc4r | melanocortin 4 receptor | 2.123 |
| 22 | Cdc42ep4 | CDC42 effector protein (Rho GTPase binding) 4 | 2.103 |
| 23 | Gabrg2 | gamma-aminobutyric acid (GABA-A) receptor. subunit gamma 2 | 2.065 |
| 24 | Acss1 | acyl-CoA synthetase short-chain family member 1 | 2.042 |
| 25 | Ttyh2 | tweety homolog 2 (Drosophila) | 2.028 |

**Supplementary Table S4: Upregulated genes in midbrain NSCs cultivated for 2 days in physioxia compared to normoxia.**

| **Midbrain NSCs (2 days) – Upregulated in 3% O_2_ (9 genes)** | | | |
| --- | --- | --- | --- |
| **No.** | **Gene symbol** | **Gene name** | **Fold change** |
| 1 | Slc16a3 | solute carrier family 16 (monocarboxylic acid transporters). member 3 | 3.873 |
| 2 | Vegfa | vascular endothelial growth factor A | 2.710 |
| 3 | Pdk1  (Q8BFP9) | pyruvate dehydrogenase kinase. isoenzyme 1 | 2.662 |
| 4 | Slc2a1 | solute carrier family 2 (facilitated glucose transporter). member 1 | 2.291 |
| 5 | P4ha1 | procollagen-proline. 2-oxoglutarate 4-dioxygenase (proline 4-hydroxylase). alpha 1 polypeptide | 2.175 |
| 6 | Egln3 | EGL nine homolog 3 (C. elegans) | 2.171 |
| 7 | Ldha | lactate dehydrogenase A | 2.135 |
| 8 | Pfkl | phosphofructokinase. liver. B-type | 2.112 |
| 9 | Tpi1 | triosephosphate isomerase 1 | 2.077 |

**Supplementary Table S5: Upregulated genes in midbrain NSCs cultivated for 13 days in physioxia compared to normoxia.**

| **Midbrain NSCs (13 days) - Upregulated in 3% O_2_ (226 genes)** | | | |  |
| --- | --- | --- | --- | --- |
| **No.** | **Gene symbol** | **Gene name** | **Fold change** | |
| 1 | Cd93 | CD93 antigen | 10.661 | |
| 2 | Col12a1 | collagen. type XII. alpha 1 | 7.459 | |
| 3 | Ier3 | immediate early response 3 | 6.929 | |
| 4 | Adamts14 | a disintegrin-like and metallopeptidase (reprolysin type) with thrombospondin type 1 motif. 14 | 5.911 | |
| 5 | Lrrc32 | leucine rich repeat containing 32 | 5.738 | |
| 6 | Apod | apolipoprotein D | 5.610 | |
| 7 | Pfkp | phosphofructokinase. platelet | 5.397 | |
| 8 | Postn | periostin. osteoblast specific factor | 5.173 | |
| 9 | Phospho1 | phosphatase. orphan 1 | 5.026 | |
| 10 | Abi3 | ABI gene family. member 3 | 5.026 | |
| 11 | Unc5b | unc-5 homolog B (C. elegans) | 4.687 | |
| 12 | Mamdc2 | MAM domain containing 2 | 4.674 | |
| 13 | Angpt2 | angiopoietin 2 | 4.668 | |
| 14 | Apln | apelin | 4.376 | |
| 15 | Nxph4 | neurexophilin 4 | 4.335 | |
| 16 | Leprel1 (P3h2. Q8CG71) | leprecan-like 1. Prolyl 3-hydroxylase 2 | 4.326 | |
| 17 | Npnt | nephronectin | 4.291 | |
| 18 | Adm | adrenomedullin | 4.241 | |
| 19 | Crispld2 | cysteine-rich secretory protein LCCL domain containing 2 | 4.230 | |
| 20 | Stc2 | stanniocalcin 2 | 4.194 | |
| 21 | Slc7a3 | solute carrier family 7 (cationic amino acid transporter. y+ system). member 3 | 4.141 | |
| 22 | Trf (Q921I1) | transferrin | 3.983 | |
| 23 | Slc2a3 | solute carrier family 2 (facilitated glucose transporter). member 3 | 3.931 | |
| 24 | Adamts1 | a disintegrin-like and metallopeptidase (reprolysin type) with thrombospondin type 1 motif. 1 | 3.874 | |
| 25 | Dhh | desert hedgehog | 3.776 | |
| 26 | Ank3 | ankyrin 3. epithelial | 3.638 | |
| 27 | Cdkn1a | cyclin-dependent kinase inhibitor 1A (P21) | 3.628 | |
| 28 | Shisa3 | shisa homolog 3 (Xenopus laevis) | 3.585 | |
| 29 | Ifitm3 | interferon induced transmembrane protein 3 | 3.517 | |
| 30 | Ndrg1 | N-myc downstream regulated gene 1 | 3.504 | |
| 31 | Tnfrsf10b | tumor necrosis factor receptor superfamily. member 10b | 3.499 | |
| 32 | Igfbp7 | insulin-like growth factor binding protein 7 | 3.475 | |
| 33 | Ctsh | cathepsin H | 3.424 | |
| 34 | Rara | retinoic acid receptor. alpha | 3.379 | |
| 35 | H2-Ab1 | histocompatibility 2. class II antigen A. beta 1 | 3.351 | |
| 36 | Camk2b | calcium/calmodulin-dependent protein kinase II. beta | 3.293 | |
| 37 | Nptx2 | neuronal pentraxin 2 | 3.243 | |
| 38 | Pdxk | pyridoxal (pyridoxine. vitamin B6) kinase | 3.239 | |
| 39 | Ndufa4l2 | NADH dehydrogenase (ubiquinone) 1 alpha subcomplex. 4-like 2 | 3.235 | |
| 40 | Adora2a | adenosine A2a receptor | 3.231 | |
| 41 | Snf1lk | SNF1-like kinase | 3.215 | |
| 42 | BC039210 (Piezo1. Q3UM62) | Piezo_RRas_bdg domain-containing protein | 3.178 | |
| 43 | Gpc3 | glypican 3 | 3.169 | |
| 44 | Ero1l | ERO1-like (S. cerevisiae) | 3.159 | |
| 45 | Kcnip1 | Kv channel-interacting protein 1 | 3.146 | |
| 46 | Bgn | biglycan | 3.144 | |
| 47 | Stc1 | stanniocalcin 1 | 3.103 | |
| 48 | Bhlhb3 | basic helix-loop-helix domain containing. class B3 | 3.088 | |
| 49 | Plp2 | proteolipid protein 2 | 3.079 | |
| 50 | Spry1 | sprouty homolog 1 (Drosophila) | similar to sprouty 1 | 3.058 | |
| 51 | Plk2 | polo-like kinase 2 (Drosophila) | 3.029 | |
| 52 | Nrp2 | neuropilin 2 | 3.010 | |
| 53 | Rab26 | RAB26. member RAS oncogene family | 3.008 | |
| 54 | Mmp14 | matrix metallopeptidase 14 (membrane-inserted) | 2.986 | |
| 55 | Cdh19 | cadherin 19. type 2 | 2.958 | |
| 56 | Nr1d1 | nuclear receptor subfamily 1. group D. member 1 | 2.905 | |
| 57 | Thra | thyroid hormone receptor alpha | 2.905 | |
| 58 | Ctsc | cathepsin C | 2.895 | |
| 59 | Ifitm2 | interferon induced transmembrane protein 2 | 2.878 | |
| 60 | Snca | synuclein. alpha | 2.878 | |
| 61 | Lrrc9 | leucine rich repeat containing 9 | 2.868 | |
| 62 | Wwc1 | WW. C2 and coiled-coil domain containing 1 | 2.859 | |
| 63 | Arpc1b | actin related protein 2/3 complex. subunit 1B | 2.856 | |
| 64 | Aox1 | aldehyde oxidase 1 | 2.848 | |
| 65 | Pfkfb3 | 6-phosphofructo-2-kinase/fructose-2.6-biphosphatase 3 | 2.846 | |
| 66 | Kcnj16 | potassium inwardly-rectifying channel. subfamily J. member 16 | 2.825 | |
| 67 | Dcn (P28654) | decorin | 2.817 | |
| 68 | Mthfd2 | methylenetetrahydrofolate dehydrogenase (NAD+ dependent). methenyltetrahydrofolate cyclohydrolase | 2.814 | |
| 69 | Atp1b1 | ATPase. Na+/K+ transporting. beta 1 polypeptide | 2.809 | |
| 70 | Ddr2 | discoidin domain receptor family. member 2 | 2.806 | |
| 71 | Gm879 (Shisa6. Q3UH99) | gene model 879. (NCBI) | 2.788 | |
| 72 | Spry4 (Q9WTP2) | sprouty homolog 4 (Drosophila) | 2.781 | |
| 73 | Car12 | carbonic anyhydrase 12 | 2.780 | |
| 74 | Kdelr3 | KDEL (Lys-Asp-Glu-Leu) endoplasmic reticulum protein retention receptor 3 | 2.771 | |
| 75 | Fkbp10 | FK506 binding protein 10 | 2.758 | |
| 76 | Aebp1 | AE binding protein 1 | 2.744 | |
| 77 | Hlf (Q8BW74) | hepatic leukemia factor | 2.742 | |
| 78 | Klf9 | Kruppel-like factor 9 | 2.710 | |
| 79 | Creb3l1 | cAMP responsive element binding protein 3-like 1 | 2.701 | |
| 80 | Scn1b | sodium channel. voltage-gated. type I. beta | 2.700 | |
| 81 | Arhgap29 | Rho GTPase activating protein 29 | 2.690 | |
| 82 | Igfbp3 | insulin-like growth factor binding protein 3 | 2.674 | |
| 83 | Adam19 | a disintegrin and metallopeptidase domain 19 (meltrin beta) | similar to metalloprotease-disintegrin meltrin beta | 2.641 | |
| 84 | Ccnd2 | cyclin D2 | 2.632 | |
| 85 | Pvr (Q8K094) | poliovirus receptor | 2.619 | |
| 86 | Gne | glucosamine | 2.616 | |
| 87 | Adora1 | adenosine A1 receptor | 2.614 | |
| 88 | Megf6 | multiple EGF-like-domains 6 | 2.612 | |
| 89 | Unc13a (Q4KUS2) | unc-13 homolog A (C. elegans) | 2.610 | |
| 90 | Loxl2 | lysyl oxidase-like 2 | 2.606 | |
| 91 | Gdf15 | growth differentiation factor 15 | 2.591 | |
| 92 | Slc12a2 | solute carrier family 12. member 2 | 2.589 | |
| 93 | Bhlhb2 | basic helix-loop-helix domain containing. class B2 | 2.576 | |
| 94 | St3gal1 | ST3 beta-galactoside alpha-2.3-sialyltransferase 1 | 2.572 | |
| 95 | Mthfd1l | methylenetetrahydrofolate dehydrogenase (NADP+ dependent) 1-like | 2.571 | |
| 96 | Tmem56 | transmembrane protein 56 | 2.552 | |
| 97 | Itga5 | integrin alpha 5 (fibronectin receptor alpha) | 2.537 | |
| 98 | Adamts9 | a disintegrin-like and metallopeptidase (reprolysin type) with thrombospondin type 1 motif. 9 | 2.536 | |
| 99 | Tubb6 | tubulin. beta 6 | 2.533 | |
| 100 | Layn | layilin | 2.527 | |
| 101 | Smpdl3b | sphingomyelin phosphodiesterase. acid-like 3B | 2.524 | |
| 102 | Raet1b | retinoic acid early transcript beta | 2.521 | |
| 103 | H60b | histocompatibility 60b | 2.521 | |
| 104 | Unc93b1 | unc-93 homolog B1 (C. elegans) | 2.514 | |
| 105 | Ecm1 | extracellular matrix protein 1 | 2.512 | |
| 106 | Slc16a3 | solute carrier family 16 (monocarboxylic acid transporters). member 3 | 2.512 | |
| 107 | Mdk | midkine | 2.512 | |
| 108 | Igfbp4 | insulin-like growth factor binding protein 4 | 2.508 | |
| 109 | Igf2 | insulin-like growth factor 2 | 2.504 | |
| 110 | Col18a1 | collagen. type XVIII. alpha 1 | 2.494 | |
| 111 | Vldlr | very low density lipoprotein receptor | 2.494 | |
| 112 | Dusp6 | dual specificity phosphatase 6 | 2.487 | |
| 113 | Tmem108 | transmembrane protein 108 | 2.485 | |
| 114 | Dusp4 | dual specificity phosphatase 4 | 2.475 | |
| 115 | Gbe1 | glucan (1.4-alpha-). branching enzyme 1 | 2.455 | |
| 116 | Vegfa | vascular endothelial growth factor A | 2.431 | |
| 117 | Tmem173 (Sting1. Q3TBT3) | transmembrane protein 173 | 2.430 | |
| 118 | Nampt | nicotinamide phosphoribosyltransferase | 2.420 | |
| 119 | Eif4ebp1 | eukaryotic translation initiation factor 4E binding protein 1 | 2.419 | |
| 120 | Cdh22 | cadherin 22 | 2.405 | |
| 121 | Slc7a1 | solute carrier family 7 (cationic amino acid transporter. y+ system). member 1 | 2.403 | |
| 122 | Zbtb7b | zinc finger and BTB domain containing 7B | 2.401 | |
| 123 | C1ql3 | C1q-like 3 | 2.393 | |
| 124 | Prss35 | protease. serine. 35 | 2.374 | |
| 125 | Pmp22 (P16646) | peripheral myelin protein | 2.363 | |
| 126 | Plagl1 | pleiomorphic adenoma gene-like 1 | 2.357 | |
| 127 | Gfpt2 | glutamine fructose-6-phosphate transaminase 2 | 2.348 | |
| 128 | Chl1 | cell adhesion molecule with homology to L1CAM | 2.344 | |
| 129 | Camk1g | calcium/calmodulin-dependent protein kinase I gamma | 2.341 | |
| 130 | Fn1 | fibronectin 1 | 2.330 | |
| 131 | Rab3b | RAB3B. member RAS oncogene family | 2.318 | |
| 132 | Egln3 | EGL nine homolog 3 (C. elegans) | 2.316 | |
| 133 | Fbn2 | fibrillin 2 | 2.314 | |
| 134 | Ccnd1 | cyclin D1 | 2.312 | |
| 135 | Stat6 | signal transducer and activator of transcription 6 | 2.310 | |
| 136 | Astn2 | astrotactin 2 | 2.303 | |
| 137 | Ptprm | protein tyrosine phosphatase. receptor type. M | 2.301 | |
| 138 | Ctsz | cathepsin Z | 2.301 | |
| 139 | Plod2 | procollagen lysine. 2-oxoglutarate 5-dioxygenase 2 | 2.288 | |
| 140 | Nudt4 | nudix (nucleoside diphosphate linked moiety X)-type motif 4 | 2.280 | |
| 141 | Phlda3 | pleckstrin homology-like domain. family A. member 3 | 2.272 | |
| 142 | Cda | cytidine deaminase | 2.269 | |
| 143 | Mrc2 | mannose receptor. C type 2 | 2.266 | |
| 144 | Oaf | OAF homolog (Drosophila) | 2.258 | |
| 145 | Hmox1 | heme oxygenase (decycling) 1 | 2.257 | |
| 146 | Lama4 | laminin. alpha 4 | 2.255 | |
| 147 | Ggta1 | glycoprotein galactosyltransferase alpha 1. 3 | 2.250 | |
| 148 | Thrsp | thyroid hormone responsive SPOT14 homolog (Rattus) | 2.242 | |
| 149 | Hif3a | hypoxia inducible factor 3. alpha subunit | 2.242 | |
| 150 | Gclc | glutamate-cysteine ligase. catalytic subunit | 2.241 | |
| 151 | St3gal5 | ST3 beta-galactoside alpha-2.3-sialyltransferase 5 | 2.238 | |
| 152 | Bag3 | Bcl2-associated athanogene 3 | 2.232 | |
| 153 | Eda2r | ectodysplasin A2 isoform receptor | 2.210 | |
| 154 | Tbc1d1 | TBC1 domain family. member 1 | 2.206 | |
| 155 | Cp | ceruloplasmin | 2.202 | |
| 156 | Bcat1 | branched chain aminotransferase 1. cytosolic | 2.201 | |
| 157 | Tm4sf1 | transmembrane 4 superfamily member 1 | 2.194 | |
| 158 | Prkcb1 | protein kinase C. beta 1 | 2.191 | |
| 159 | Prelid2 | PRELI domain containing 2 | 2.190 | |
| 160 | Emcn | endomucin | 2.186 | |
| 161 | Hk2 | hexokinase 2 | 2.186 | |
| 162 | Abcc1 | ATP-binding cassette. sub-family C (CFTR/MRP). member 1 | 2.175 | |
| 163 | Ajap1 | adherens junction associated protein 1 | 2.167 | |
| 164 | Kcnma1 | potassium large conductance calcium-activated channel. subfamily M. alpha member 1 | 2.158 | |
| 165 | Cdca7l | cell division cycle associated 7 like | 2.156 | |
| 166 | Tox2 | TOX high mobility group box family member 2 | 2.146 | |
| 167 | Ephx1 | epoxide hydrolase 1. microsomal | 2.146 | |
| 168 | Adam12 | a disintegrin and metallopeptidase domain 12 (meltrin alpha) | 2.144 | |
| 169 | Ppp1r3g | protein phosphatase 1. regulatory (inhibitor) subunit 3G | 2.141 | |
| 170 | Slc35c2 | solute carrier family 35. member C2 | 2.138 | |
| 171 | Etv5 | ets variant gene 5 | 2.126 | |
| 172 | Crtap | cartilage associated protein | 2.126 | |
| 173 | Chrm3 | cholinergic receptor. muscarinic 3. cardiac | 2.126 | |
| 174 | Bmp7 | bone morphogenetic protein 7 | 2.125 | |
| 175 | Akap12 | A kinase (PRKA) anchor protein (gravin) 12 | 2.120 | |
| 176 | Cox4i2 | cytochrome c oxidase subunit IV isoform 2 | 2.109 | |
| 177 | Maml3 | mastermind like 3 (Drosophila) | 2.109 | |
| 178 | Slc43a3 | solute carrier family 43. member 3 | 2.106 | |
| 179 | Id1 | inhibitor of DNA binding 1 | 2.105 | |
| 180 | Itga1 | integrin alpha 1 | 2.103 | |
| 181 | Etv4 | ets variant gene 4 (E1A enhancer binding protein. E1AF) | 2.098 | |
| 182 | Kcnn4 | potassium intermediate/small conductance calcium-activated channel. subfamily N. member 4 | 2.098 | |
| 183 | Flnc | filamin C. gamma (actin binding protein 280) | 2.097 | |
| 184 | Slc41a2 | solute carrier family 41. member 2 | 2.094 | |
| 185 | Hdac9 | histone deacetylase 9 | 2.093 | |
| 186 | Odz4 | odd Oz/ten-m homolog 4 (Drosophila) | 2.086 | |
| 187 | Synj2 | synaptojanin 2 | 2.085 | |
| 188 | Ttpa | tocopherol (alpha) transfer protein | 2.080 | |
| 189 | Frmd3 | FERM domain containing 3 | 2.078 | |
| 190 | Mical2 | microtubule associated monoxygenase. calponin and LIM domain containing 2 | 2.073 | |
| 191 | Oxr1 | oxidation resistance 1 | 2.072 | |
| 192 | Dusp1 | dual specificity phosphatase 1 | 2.071 | |
| 193 | Lamb1-1 | laminin B1 subunit 1 | 2.071 | |
| 194 | Nrn1 | neuritin 1 | 2.068 | |
| 195 | Ramp1 | receptor (calcitonin) activity modifying protein 1 | 2.067 | |
| 196 | Fgf11 | fibroblast growth factor 11 | 2.066 | |
| 197 | Ldlr | low density lipoprotein receptor | 2.061 | |
| 198 | H2-K1 | histocompatibility 2. K1. K region | 2.060 | |
| 199 | H2-Q6 | histocompatibility 2. Q region locus 6 | 2.059 | |
| 200 | Tfrc | transferrin receptor | 2.058 | |
| 201 | Smad3 | MAD homolog 3 (Drosophila) | 2.056 | |
| 202 | Tbc1d8b | TBC1 domain family. member 8B | 2.056 | |
| 203 | Hs6st2 | heparan sulfate 6-O-sulfotransferase 2 | 2.050 | |
| 204 | Znrf2 | zinc and ring finger 2 | 2.050 | |
| 205 | Papln | papilin. proteoglycan-like sulfated glycoprotein | 2.050 | |
| 206 | Osbpl10 | oxysterol binding protein-like 10 | 2.049 | |
| 207 | Nwd1 | NACHT and WD repeat domain containing 1 | 2.048 | |
| 208 | Tmem38a | transmembrane protein 38A | 2.048 | |
| 209 | Impa2 | inositol (myo)-1(or 4)-monophosphatase 2 | 2.044 | |
| 210 | Cacna1h | calcium channel. voltage-dependent. T type. alpha 1H subunit | 2.043 | |
| 211 | Camkk2 | calcium/calmodulin-dependent protein kinase kinase 2. beta | 2.042 | |
| 212 | Polh | polymerase (DNA directed). eta (RAD 30 related) | 2.041 | |
| 213 | Nomo1 | nodal modulator 1 | 2.034 | |
| 214 | Pcdh20 | protocadherin 20 | 2.034 | |
| 215 | Kirrel3 | kin of IRRE like 3 (Drosophila) | 2.031 | |
| 216 | Chst7 | carbohydrate (N-acetylglucosamino) sulfotransferase 7 | 2.029 | |
| 217 | Chst11 | carbohydrate sulfotransferase 11 | 2.029 | |
| 218 | Ubash3b | ubiquitin associated and SH3 domain containing. B | 2.028 | |
| 219 | Cyba | cytochrome b-245. alpha polypeptide | 2.021 | |
| 220 | Peg10 | paternally expressed 10 | 2.016 | |
| 221 | Hrh1 | histamine receptor H1 | 2.013 | |
| 222 | Olfm2 | olfactomedin 2 | 2.010 | |
| 223 | Mef2c | myocyte enhancer factor 2C | 2.008 | |
| 224 | Rrp1b | ribosomal RNA processing 1 homolog B (S. cerevisiae) | 2.006 | |
| 225 | Crim1 | cysteine rich transmembrane BMP regulator 1 (chordin like) | 2.003 | |
| 226 | Gpr162 | G protein-coupled receptor 162 | 2.003 | |

**Supplementary Table S6: Downregulated genes in midbrain NSCs cultivated for 13 days in physiooxia compared to normoxia.**

| **Midbrain 13d - Downregulated in 3% O_2_ (112 genes)** | | | |  |
| --- | --- | --- | --- | --- |
| **No.** | **Gene symbol** | **Gene name** | **Fold change** | |
| 1 | Gje1 (Q9CX92) | gap junction membrane channel protein epsilon 1 | 6.414 | |
| 2 | Gpr17 | G protein-coupled receptor 17 | 4.704 | |
| 3 | Gabbr2 | gamma-aminobutyric acid (GABA) B receptor 2 | 4.516 | |
| 4 | Actc1 | actin. alpha. cardiac | 4.329 | |
| 5 | Sncaip | synuclein. alpha interacting protein (synphilin) | 4.200 | |
| 6 | Prkcq | protein kinase C. theta | 4.136 | |
| 7 | Abca1 | ATP-binding cassette. sub-family A (ABC1). member 1 | 4.005 | |
| 8 | Slc24a3 | solute carrier family 24 (sodium/potassium/calcium exchanger). member 3 | 3.820 | |
| 9 | Cyp4f15 | cytochrome P450. family 4. subfamily f. polypeptide 15 | 3.723 | |
| 10 | Mc5r | melanocortin 5 receptor | 3.605 | |
| 11 | Naaa | Nacylethanolamine acid amidase | 3.565 | |
| 12 | 6330403K07Rik | RIKEN cDNA 6330403K07 gene | 3.477 | |
| 13 | Cyp2j9 | cytochrome P450. family 2. subfamily j. polypeptide 9 | 3.272 | |
| 14 | Lrrtm2 | leucine rich repeat transmembrane neuronal 2 | 3.200 | |
| 15 | Dll3 | delta-like 3 (Drosophila) | 3.155 | |
| 16 | Nkain4 | Na+/K+ transporting ATPase interacting 4 | 3.097 | |
| 17 | Rnd3 | Rho family GTPase 3 | 3.078 | |
| 18 | Cdh8 | cadherin 8 | 3.071 | |
| 19 | Lypd1 | Ly6/Plaur domain containing 1 | 3.064 | |
| 20 | Igsf11 | immunoglobulin superfamily. member 11 | 3.022 | |
| 21 | Myt1 (Q8CFC2) | myelin transcription factor 1 | 3.012 | |
| 22 | Omg | oligodendrocyte myelin glycoprotein | 2.974 | |
| 23 | Pak3 | p21 (CDKN1A)-activated kinase 3 | 2.969 | |
| 24 | Gm687 | gene model 687. (NCBI) | 2.943 | |
| 25 | Dmrta1 | doublesex and mab-3 related transcription factor like family A1 | 2.918 | |
| 26 | Nrbp2 | nuclear receptor binding protein 2 | 2.886 | |
| 27 | Hes5 | hairy and enhancer of split 5 (Drosophila) | 2.881 | |
| 28 | Pde1b | phosphodiesterase 1B. Ca2+-calmodulin dependent | 2.865 | |
| 29 | Tbc1d4 | TBC1 domain family. member 4 | 2.856 | |
| 30 | Kcnn2 | potassium intermediate/small conductance calcium-activated channel. subfamily N. member 2 | 2.846 | |
| 31 | Kit | kit oncogene | 2.791 | |
| 32 | Abat | 4-aminobutyrate aminotransferase | 2.779 | |
| 33 | Gjd2 | gap junction protein. delta 2 | 2.731 | |
| 34 | Gdap1l1 | ganglioside-induced differentiation-associated protein 1-like 1 | 2.724 | |
| 35 | Plxnb3 | plexin B3 | 2.674 | |
| 36 | Lipg (Q9WVG5) | lipase. endothelial | 2.648 | |
| 37 | Tenc1 | tensin like C1 domain-containing phosphatase | 2.634 | |
| 38 | Gdpd2 | glycerophosphodiester phosphodiesterase domain containing 2 | 2.611 | |
| 39 | Apcdd1 | adenomatosis polyposis coli down-regulated 1 | 2.599 | |
| 40 | Dcc | deleted in colorectal carcinoma | 2.594 | |
| 41 | Vcam1 | vascular cell adhesion molecule 1 | 2.591 | |
| 42 | Enpp2 | ectonucleotide pyrophosphatase/phosphodiesterase 2 | 2.583 | |
| 43 | Punc | putative neuronal cell adhesion molecule | 2.567 | |
| 44 | Cd82 | CD82 antigen | 2.534 | |
| 45 | Rap1gap | Rap1 GTPase-activating protein | 2.528 | |
| 46 | Adhfe1 | alcohol dehydrogenase. iron containing. 1 | 2.528 | |
| 47 | Mapk8ip2 | mitogen-activated protein kinase 8 interacting protein 2 | 2.524 | |
| 48 | Celsr1 | cadherin. EGF LAG seven-pass G-type receptor 1 (flamingo homolog. Drosophila) | 2.497 | |
| 49 | Ina | internexin neuronal intermediate filament protein. alpha | 2.485 | |
| 50 | Adcy8 | adenylate cyclase 8 | 2.469 | |
| 51 | Il18 | interleukin 18 | 2.465 | |
| 52 | Epha4 | Eph receptor A4 | 2.459 | |
| 53 | Frzb | frizzled-related protein | 2.452 | |
| 54 | Pcx | pyruvate carboxylase | 2.429 | |
| 55 | Pde9a | phosphodiesterase 9A | 2.399 | |
| 56 | Txnip | thioredoxin interacting protein | 2.395 | |
| 57 | Acss1 | acyl-CoA synthetase short-chain family member 1 | 2.373 | |
| 58 | Lrp2 | low density lipoprotein receptor-related protein 2 | 2.363 | |
| 59 | Tbx15 | T-box 15 | 2.339 | |
| 60 | Gca | grancalcin | 2.327 | |
| 61 | Mfsd6 | major facilitator superfamily domain containing 6 | 2.327 | |
| 62 | Ppp2r2b | protein phosphatase 2 (formerly 2A). regulatory subunit B (PR 52). beta isoform | 2.321 | |
| 63 | Scg3 | secretogranin III | 2.319 | |
| 64 | Rhbdl3 | rhomboid. veinlet-like 3 (Drosophila) | 2.318 | |
| 65 | Mfng | MFNG O-fucosylpeptide 3-beta-N-acetylglucosaminyltransferase | 2.315 | |
| 66 | Gdap1 | ganglioside-induced differentiation-associated-protein 1 | 2.297 | |
| 67 | Fmo1 | flavin containing monooxygenase 1 | 2.295 | |
| 68 | Nrarp | Notch-regulated ankyrin repeat protein | 2.290 | |
| 69 | Lrrn1 | leucine rich repeat protein 1. neuronal | 2.284 | |
| 70 | Plxnc1 | plexin C1 | 2.264 | |
| 71 | Atp10b | ATPase. class V. type 10B | 2.258 | |
| 72 | Plcd4 | phospholipase C. delta 4 | 2.257 | |
| 73 | Fbp1 (Q9QXD6) | fructose bisphosphatase 1 | 2.250 | |
| 74 | Pdpn | podoplanin | 2.228 | |
| 75 | Meig1 | meiosis expressed gene 1 | 2.222 | |
| 76 | Tcp11l2 | t-complex 11 (mouse) like 2 | 2.218 | |
| 77 | Id4 | inhibitor of DNA binding 4 | 2.210 | |
| 78 | Ttyh1 | tweety homolog 1 (Drosophila) | 2.209 | |
| 79 | Ttyh2 | tweety homolog 2 (Drosophila) | 2.205 | |
| 80 | Epb4.1 | erythrocyte protein band 4.1 | 2.200 | |
| 81 | Abhd3 | abhydrolase domain containing 3 | 2.199 | |
| 82 | Tspan15 | tetraspanin 15 | 2.189 | |
| 83 | Chrna3 | cholinergic receptor. nicotinic. alpha polypeptide 3 | 2.187 | |
| 84 | Zfp488 | zinc finger protein 488 | 2.171 | |
| 85 | Mc4r | melanocortin 4 receptor | 2.156 | |
| 86 | Tle1 | transducin-like enhancer of split 1. homolog of Drosophila E(spl) | 2.138 | |
| 87 | Amotl2 | angiomotin like 2 | 2.138 | |
| 88 | Dll1 | delta-like 1 (Drosophila) | 2.132 | |
| 89 | Slitrk1 | SLIT and NTRK-like family. member 1 | 2.131 | |
| 90 | Lpar4 | lysophosphatidic acid receptor 4 | 2.125 | |
| 91 | Gm967 | gene model 967. (NCBI) | 2.108 | |
| 92 | Pmepa1 | prostate transmembrane protein. androgen induced 1 | 2.107 | |
| 93 | Sfrp5 | secreted frizzled-related sequence protein 5 | 2.103 | |
| 94 | Paqr3 | progestin and adipoQ receptor family member III | 2.098 | |
| 95 | Gadd45g | growth arrest and DNA-damage-inducible 45 gamma | 2.088 | |
| 96 | Ap1s2 | adaptor-related protein complex 1. sigma 2 subunit | 2.088 | |
| 97 | Arrdc4 | arrestin domain containing 4 | 2.083 | |
| 98 | Calr4 | calreticulin 4 | 2.079 | |
| 99 | Rnd2 | Rho family GTPase 2 | 2.079 | |
| 100 | BC034069 |  | 2.074 | |
| 101 | Lrrc4 | leucine rich repeat containing 4 | 2.073 | |
| 102 | Hsdl2 | hydroxysteroid dehydrogenase like 2 | 2.045 | |
| 103 | Tox | thymocyte selection-associated high mobility group box | similar to thymus high mobility group box protein TOX | 2.036 | |
| 104 | Enpp3 | ectonucleotide pyrophosphatase/phosphodiesterase 3 | 2.021 | |
| 105 | Ap3b2 | adaptor-related protein complex 3. beta 2 subunit | 2.019 | |
| 106 | Tst | thiosulfate sulfurtransferase. mitochondrial | 2.013 | |
| 107 | Ccbl2 | cysteine conjugate-beta lyase 2 | 2.012 | |
| 108 | Bcl2 | B-cell leukemia/lymphoma 2 | similar to Bcl2-like protein | 2.012 | |
| 109 | Asrgl1 | asparaginase like 1 | 2.009 | |
| 110 | Nkd1 | naked cuticle 1 homolog (Drosophila) | 2.009 | |
| 111 | Btg2 | B-cell translocation gene 2. anti-proliferative | 2.008 | |
| 112 | Pkia | protein kinase inhibitor. alpha | 2.007 | |

**Supplementary Table S7: Affected biological processes. molecular functions and components from upregulated genes in midbrain and cortical NSCs cultivated for 2 days in physioxia.**

| **Midbrain NSCs** | **Cortex NSCs** |
| --- | --- |
| | [**GO biological process**](http://pantherdb.org/tools/compareToRefList.jsp?sortOrder=1&sortList=categories)  *Mus musculus* (reference) | [Number](http://pantherdb.org/tools/compareToRefList.jsp?sortOrder=2&sortList=upload_1&sortField=num) of genes | [Fold Enrichment](http://pantherdb.org/tools/compareToRefList.jsp?sortOrder=2&sortList=upload_1&sortField=foldEnrich) | [P value](http://pantherdb.org/tools/compareToRefList.jsp?sortOrder=1&sortList=upload_1&sortField=pval) | | --- | --- | --- | --- | | [pyruvate metabolic process](http://amigo.geneontology.org/amigo/term/GO:0006090) | [3](http://pantherdb.org/tools/gxIdsList.do?acc=GO:0006090&list=upload_1&organism=Mus musculus) | > 100 | 2.62E-02 | | [glucose metabolic process](http://amigo.geneontology.org/amigo/term/GO:0006006) | [4](http://pantherdb.org/tools/gxIdsList.do?acc=GO:0006006&list=upload_1&organism=Mus musculus) | > 100 | 4.13E-04 | | 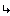 [hexose metabolic process](http://amigo.geneontology.org/amigo/term/GO:0019318) | [4](http://pantherdb.org/tools/gxIdsList.do?acc=GO:0019318&list=upload_1&organism=Mus musculus) | 78.18 | 1.25E-03 | | 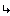 [monosaccharide metabolic process](http://amigo.geneontology.org/amigo/term/GO:0005996) | [4](http://pantherdb.org/tools/gxIdsList.do?acc=GO:0005996&list=upload_1&organism=Mus musculus) | 68.34 | 2.12E-03 | | [carbohydrate catabolic process](http://amigo.geneontology.org/amigo/term/GO:0016052) | [3](http://pantherdb.org/tools/gxIdsList.do?acc=GO:0016052&list=upload_1&organism=Mus musculus) | 86.23 | 4.60E-02 | | [cellular response to hypoxia](http://amigo.geneontology.org/amigo/term/GO:0071456) | [3](http://pantherdb.org/tools/gxIdsList.do?acc=GO:0071456&list=upload_1&organism=Mus musculus) | 85.22 | 4.76E-02 | |  |  |  |  | | | [**GO biological process**](http://pantherdb.org/tools/compareToRefList.jsp?sortOrder=1&sortList=categories)  *Mus musculus* (reference) | [Number](http://pantherdb.org/tools/compareToRefList.jsp?sortOrder=2&sortList=upload_1&sortField=num) of genes | [Fold Enrichment](http://pantherdb.org/tools/compareToRefList.jsp?sortOrder=2&sortList=upload_1&sortField=foldEnrich) | [P value](http://pantherdb.org/tools/compareToRefList.jsp?sortOrder=1&sortList=upload_1&sortField=pval) | | --- | --- | --- | --- | | [peptidyl-proline hydroxylation to 4-hydroxy-L-proline](http://amigo.geneontology.org/amigo/term/GO:0018401) | [4](http://pantherdb.org/tools/gxIdsList.do?acc=GO:0018401&list=upload_1&organism=Mus musculus) | > 100 | 5.75E-05 | | 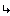 [peptidyl-proline hydroxylation](http://amigo.geneontology.org/amigo/term/GO:0019511) | [4](http://pantherdb.org/tools/gxIdsList.do?acc=GO:0019511&list=upload_1&organism=Mus musculus) | > 100 | 1.29E-04 | | 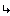 [peptidyl-proline modification](http://amigo.geneontology.org/amigo/term/GO:0018208) | [4](http://pantherdb.org/tools/gxIdsList.do?acc=GO:0018208&list=upload_1&organism=Mus musculus) | 68.07 | 4.58E-03 | | 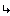 [protein hydroxylation](http://amigo.geneontology.org/amigo/term/GO:0018126) | [4](http://pantherdb.org/tools/gxIdsList.do?acc=GO:0018126&list=upload_1&organism=Mus musculus) | 89.20 | 1.69E-03 | | [cellular response to hypoxia](http://amigo.geneontology.org/amigo/term/GO:0071456) | [5](http://pantherdb.org/tools/gxIdsList.do?acc=GO:0071456&list=upload_1&organism=Mus musculus) | 37.60 | 2.46E-03 | | 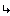 [response to hypoxia](http://amigo.geneontology.org/amigo/term/GO:0001666) | [7](http://pantherdb.org/tools/gxIdsList.do?acc=GO:0001666&list=upload_1&organism=Mus musculus) | 23.33 | 1.87E-04 | | 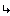 [response to decreased oxygen levels](http://amigo.geneontology.org/amigo/term/GO:0036293) | [7](http://pantherdb.org/tools/gxIdsList.do?acc=GO:0036293&list=upload_1&organism=Mus musculus) | 22.52 | 2.37E-04 | | 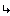 [response to oxygen levels](http://amigo.geneontology.org/amigo/term/GO:0070482) | [7](http://pantherdb.org/tools/gxIdsList.do?acc=GO:0070482&list=upload_1&organism=Mus musculus) | 20.86 | 3.94E-04 | | 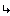 [cellular response to decreased oxygen levels](http://amigo.geneontology.org/amigo/term/GO:0036294) | [5](http://pantherdb.org/tools/gxIdsList.do?acc=GO:0036294&list=upload_1&organism=Mus musculus) | 35.53 | 3.21E-03 | | 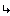 [cellular response to oxygen levels](http://amigo.geneontology.org/amigo/term/GO:0071453) | [5](http://pantherdb.org/tools/gxIdsList.do?acc=GO:0071453&list=upload_1&organism=Mus musculus) | 30.50 | 6.63E-03 | | [glucose metabolic process](http://amigo.geneontology.org/amigo/term/GO:0006006) | [5](http://pantherdb.org/tools/gxIdsList.do?acc=GO:0006006&list=upload_1&organism=Mus musculus) | 34.40 | 3.75E-03 | | 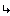 [hexose metabolic process](http://amigo.geneontology.org/amigo/term/GO:0019318) | [5](http://pantherdb.org/tools/gxIdsList.do?acc=GO:0019318&list=upload_1&organism=Mus musculus) | 25.87 | 1.45E-02 | | 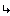 [monosaccharide metabolic process](http://amigo.geneontology.org/amigo/term/GO:0005996) | [5](http://pantherdb.org/tools/gxIdsList.do?acc=GO:0005996&list=upload_1&organism=Mus musculus) | 22.61 | 2.75E-02 | |
|  |  |
| | **GO molecular function**  [*Mus musculus*](http://pantherdb.org/tools/gxIdsList.do?reflist=1) (reference) | [Number](http://pantherdb.org/tools/compareToRefList.jsp?sortOrder=2&sortList=upload_1&sortField=num) of genes | [Fold Enrichment](http://pantherdb.org/tools/compareToRefList.jsp?sortOrder=2&sortList=upload_1&sortField=foldEnrich) | [P value](http://pantherdb.org/tools/compareToRefList.jsp?sortOrder=1&sortList=upload_1&sortField=pval) | | | --- | --- | --- | --- | --- | | [peptidyl-proline 4-dioxygenase activity](http://amigo.geneontology.org/amigo/term/GO:0031545) | [2](http://pantherdb.org/tools/gxIdsList.do?acc=GO:0031545&list=upload_1&organism=Mus musculus) | > 100 | 1.89E-02 | | | 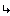 [peptidyl-proline dioxygenase activity](http://amigo.geneontology.org/amigo/term/GO:0031543) | [2](http://pantherdb.org/tools/gxIdsList.do?acc=GO:0031543&list=upload_1&organism=Mus musculus) | > 100 | 3.82E-02 | | | [monosaccharide binding](http://amigo.geneontology.org/amigo/term/GO:0048029) | [3](http://pantherdb.org/tools/gxIdsList.do?acc=GO:0048029&list=upload_1&organism=Mus musculus) | 86.23 | 1.44E-02 | | | [identical protein binding](http://amigo.geneontology.org/amigo/term/GO:0042802) | [7](http://pantherdb.org/tools/gxIdsList.do?acc=GO:0042802&list=upload_1&organism=Mus musculus) | 7.82 | 8.23E-03 | | |  |  |  | |  | | | [**GO molecular function**](http://pantherdb.org/tools/compareToRefList.jsp?sortOrder=1&sortList=categories)  *Mus musculus* (reference) | [Number](http://pantherdb.org/tools/compareToRefList.jsp?sortOrder=2&sortList=upload_1&sortField=num) of genes | [Fold Enrichment](http://pantherdb.org/tools/compareToRefList.jsp?sortOrder=2&sortList=upload_1&sortField=foldEnrich) | [P value](http://pantherdb.org/tools/compareToRefList.jsp?sortOrder=1&sortList=upload_1&sortField=pval) | | --- | --- | --- | --- | | [phosphofructokinase activity](http://amigo.geneontology.org/amigo/term/GO:0008443) | [3](http://pantherdb.org/tools/gxIdsList.do?acc=GO:0008443&list=upload_1&organism=Mus musculus) | > 100 | 1.13E-03 | | 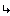 [carbohydrate kinase activity](http://amigo.geneontology.org/amigo/term/GO:0019200) | [3](http://pantherdb.org/tools/gxIdsList.do?acc=GO:0019200&list=upload_1&organism=Mus musculus) | 92.39 | 1.88E-02 | | [peptidyl-proline 4-dioxygenase activity](http://amigo.geneontology.org/amigo/term/GO:0031545) | [3](http://pantherdb.org/tools/gxIdsList.do?acc=GO:0031545&list=upload_1&organism=Mus musculus) | > 100 | 1.55E-03 | | 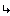 [peptidyl-proline dioxygenase activity](http://amigo.geneontology.org/amigo/term/GO:0031543) | [3](http://pantherdb.org/tools/gxIdsList.do?acc=GO:0031543&list=upload_1&organism=Mus musculus) | > 100 | 4.27E-03 | | 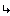 [2-oxoglutarate-dependent dioxygenase activity](http://amigo.geneontology.org/amigo/term/GO:0016706) | [4](http://pantherdb.org/tools/gxIdsList.do?acc=GO:0016706&list=upload_1&organism=Mus musculus) | 56.24 | 2.93E-03 | | 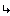 [dioxygenase activity](http://amigo.geneontology.org/amigo/term/GO:0051213) | [5](http://pantherdb.org/tools/gxIdsList.do?acc=GO:0051213&list=upload_1&organism=Mus musculus) | 35.15 | 1.06E-03 | | 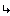 [oxidoreductase activity](http://amigo.geneontology.org/amigo/term/GO:0016491) | [9](http://pantherdb.org/tools/gxIdsList.do?acc=GO:0016491&list=upload_1&organism=Mus musculus) | 7.33 | 7.11E-03 | | [L-ascorbic acid binding](http://amigo.geneontology.org/amigo/term/GO:0031418) | [3](http://pantherdb.org/tools/gxIdsList.do?acc=GO:0031418&list=upload_1&organism=Mus musculus) | > 100 | 1.43E-02 | | 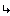 [monosaccharide binding](http://amigo.geneontology.org/amigo/term/GO:0048029) | [6](http://pantherdb.org/tools/gxIdsList.do?acc=GO:0048029&list=upload_1&organism=Mus musculus) | 45.65 | 1.46E-05 | | 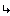 [carbohydrate binding](http://amigo.geneontology.org/amigo/term/GO:0030246) | [7](http://pantherdb.org/tools/gxIdsList.do?acc=GO:0030246&list=upload_1&organism=Mus musculus) | 16.52 | 5.79E-04 | | [iron ion binding](http://amigo.geneontology.org/amigo/term/GO:0005506) | [5](http://pantherdb.org/tools/gxIdsList.do?acc=GO:0005506&list=upload_1&organism=Mus musculus) | 19.48 | 1.74E-02 | |
|  |  |
|  | | [**GO cellular component**](http://pantherdb.org/tools/compareToRefList.jsp?sortOrder=1&sortList=categories)  *Mus musculus* (reference) | [Number](http://pantherdb.org/tools/compareToRefList.jsp?sortOrder=2&sortList=upload_1&sortField=num) of genes | [Fold Enrichment](http://pantherdb.org/tools/compareToRefList.jsp?sortOrder=2&sortList=upload_1&sortField=foldEnrich) | [P value](http://pantherdb.org/tools/compareToRefList.jsp?sortOrder=1&sortList=upload_1&sortField=pval) | | --- | --- | --- | --- | | [procollagen-proline 4-dioxygenase complex](http://amigo.geneontology.org/amigo/term/GO:0016222) | [2](http://pantherdb.org/tools/gxIdsList.do?acc=GO:0016222&list=upload_1&organism=Mus musculus) | > 100 | 3.36E-02 | | [6-phosphofructokinase complex](http://amigo.geneontology.org/amigo/term/GO:0005945) | [2](http://pantherdb.org/tools/gxIdsList.do?acc=GO:0005945&list=upload_1&organism=Mus musculus) | > 100 | 3.36E-02 | |  |  |  |  | |

**Supplementary Table S8: Affected biological processes. molecular functions and components from upregulated genes in midbrain and cortical NSCs cultivated for 13 days in physioxia.**

| **Midbrain NSCs** | **Cortex NSCs** |
| --- | --- |
| | [**GO biological process**](http://pantherdb.org/tools/compareToRefList.jsp?sortOrder=1&sortList=categories)  *Mus musculus* (reference) | [Number](http://pantherdb.org/tools/compareToRefList.jsp?sortOrder=2&sortList=upload_1&sortField=num) of genes | [Fold Enrichment](http://pantherdb.org/tools/compareToRefList.jsp?sortOrder=2&sortList=upload_1&sortField=foldEnrich) | [P value](http://pantherdb.org/tools/compareToRefList.jsp?sortOrder=1&sortList=upload_1&sortField=pval) | | --- | --- | --- | --- | | protein hydroxylation | 6 | 20.13 | 1.19E-02 | | 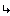 cellular process | 194 | 1.25 | 2.97E-05 | | [positive regulation of angiogenesis](http://amigo.geneontology.org/amigo/term/GO:0045766) | 12 | 6.91 | 3.25E-03 | | 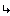 [regulation of angiogenesis](http://amigo.geneontology.org/amigo/term/GO:0045765) | 20 | 6.69 | 6.38E-07 | | 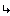 [regulation of anatomical structure morphogenesis](http://amigo.geneontology.org/amigo/term/GO:0022603) | 33 | 3.32 | 2.30E-05 | | 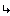 [regulation of developmental process](http://amigo.geneontology.org/amigo/term/GO:0050793) | 67 | 2.59 | 1.86E-09 | | 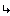 [regulation of biological process](http://amigo.geneontology.org/amigo/term/GO:0050789) | 166 | 1.35 | 6.85E-05 | | 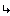 [biological regulation](http://amigo.geneontology.org/amigo/term/GO:0065007) | 172 | 1.33 | 3.80E-05 | | 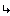 [regulation of vasculature development](http://amigo.geneontology.org/amigo/term/GO:1901342) | 20 | 6.60 | 8.01E-07 | | 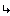 [regulation of multicellular organismal development](http://amigo.geneontology.org/amigo/term/GO:2000026) | 47 | 3.16 | 2.55E-08 | | 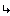 [regulation of multicellular organismal process](http://amigo.geneontology.org/amigo/term/GO:0051239) | 78 | 2.72 | 4.19E-13 | | 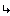 [positive regulation of vasculature development](http://amigo.geneontology.org/amigo/term/GO:1904018) | 12 | 6.91 | 3.25E-03 | | 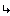 [positive regulation of multicellular organismal process](http://amigo.geneontology.org/amigo/term/GO:0051240) | 43 | 2.67 | 4.01E-05 | | 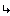 [positive regulation of biological process](http://amigo.geneontology.org/amigo/term/GO:0048518) | 116 | 1.83 | 2.67E-09 | | 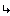 [positive regulation of developmental process](http://amigo.geneontology.org/amigo/term/GO:0051094) | 40 | 2.74 | 7.69E-05 | | [extracellular matrix organization](http://amigo.geneontology.org/amigo/term/GO:0030198) | 18 | 6.53 | 8.35E-06 | | 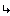 [extracellular structure organization](http://amigo.geneontology.org/amigo/term/GO:0043062) | 18 | 6.51 | 8.83E-06 | | 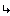 external encapsulating structure organization | 18 | 6.51 | 8.83E-06 | | [regulation of endothelial cell migration](http://amigo.geneontology.org/amigo/term/GO:0010594) | 11 | 6.33 | 2.24E-02 | | 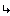 [regulation of cell migration](http://amigo.geneontology.org/amigo/term/GO:0030334) | 33 | 3.42 | 1.14E-05 | | 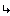 [regulation of cell motility](http://amigo.geneontology.org/amigo/term/GO:2000145) | 34 | 3.34 | 1.07E-05 | | 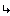 [regulation of cellular component movement](http://amigo.geneontology.org/amigo/term/GO:0051270) | 35 | 3.16 | 2.32E-05 | | 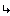 [regulation of localization](http://amigo.geneontology.org/amigo/term/GO:0032879) | 68 | 2.33 | 2.11E-07 | | 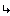 [regulation of cellular process](http://amigo.geneontology.org/amigo/term/GO:0050794) | 163 | 1.38 | 1.42E-05 | | 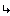 [regulation of locomotion](http://amigo.geneontology.org/amigo/term/GO:0040012) | 36 | 3.37 | 2.50E-06 | | [ossification](http://amigo.geneontology.org/amigo/term/GO:0001503) | 14 | 6.03 | 1.66E-03 | | 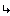 [multicellular organismal process](http://amigo.geneontology.org/amigo/term/GO:0032501) | 121 | 1.61 | 5.64E-06 | | [response to hypoxia](http://amigo.geneontology.org/amigo/term/GO:0001666) | 12 | 6.02 | 1.29E-02 | | 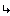 [response to decreased oxygen levels](http://amigo.geneontology.org/amigo/term/GO:0036293) | 12 | 5.81 | 1.82E-02 | |  [response to oxygen levels](http://amigo.geneontology.org/amigo/term/GO:0070482) | 12 | 5.38 | 3.86E-02 | | [response to abiotic stimulus](http://amigo.geneontology.org/amigo/term/GO:0009628) | 27 | 2.80 | 1.85E-02 | | [negative regulation of neuron apoptotic process](http://amigo.geneontology.org/amigo/term/GO:0043524) | 11 | 5.85 | 4.60E-02 | | [negative regulation of apoptotic process](http://amigo.geneontology.org/amigo/term/GO:0043066) | 30 | 3.17 | 3.52E-04 | | [regulation of apoptotic process](http://amigo.geneontology.org/amigo/term/GO:0042981) | 46 | 3.05 | 1.53E-07 | | [regulation of programmed cell death](http://amigo.geneontology.org/amigo/term/GO:0043067) | 46 | 2.98 | 3.39E-07 | | [regulation of cell death](http://amigo.geneontology.org/amigo/term/GO:0010941) | 48 | 2.80 | 8.77E-07 | | [negative regulation of programmed cell death](http://amigo.geneontology.org/amigo/term/GO:0043069) | 30 | 3.10 | 5.69E-04 | | [negative regulation of cell death](http://amigo.geneontology.org/amigo/term/GO:0060548) | 32 | 2.93 | 6.65E-04 | | [negative regulation of cellular process](http://amigo.geneontology.org/amigo/term/GO:0048523) | 99 | 2.00 | 3.04E-09 | | [negative regulation of biological process](http://amigo.geneontology.org/amigo/term/GO:0048519) | 109 | 2.01 | 3.07E-11 | | [regulation of neuron apoptotic process](http://amigo.geneontology.org/amigo/term/GO:0043523) | 15 | 5.41 | 2.27E-03 | | [regulation of neuron death](http://amigo.geneontology.org/amigo/term/GO:1901214) | 17 | 4.38 | 6.12E-03 | | [vascular process in circulatory system](http://amigo.geneontology.org/amigo/term/GO:0003018) | 13 | 5.75 | 7.58E-03 | | [circulatory system process](http://amigo.geneontology.org/amigo/term/GO:0003013) | 21 | 4.62 | 1.15E-04 | | regulation of cell-substrate adhesion | 12 | 5.28 | 4.61E-02 | | [regulation of cell adhesion](http://amigo.geneontology.org/amigo/term/GO:0030155) | 28 | 3.72 | 4.01E-05 | | [negative regulation of cell adhesion](http://amigo.geneontology.org/amigo/term/GO:0007162) | 15 | 4.98 | 6.09E-03 | | [blood circulation](http://amigo.geneontology.org/amigo/term/GO:0008015) | 20 | 4.73 | 1.78E-04 | | regulation of cellular response to growth factor stimulus | 14 | 4.73 | 2.57E-02 | | regulation of response to stimulus | 81 | 2.02 | 1.12E-06 | | [regulation of epithelial cell proliferation](http://amigo.geneontology.org/amigo/term/GO:0050678) | 17 | 4.41 | 5.51E-03 | | [regulation of cell population proliferation](http://amigo.geneontology.org/amigo/term/GO:0042127) | 50 | 2.86 | 1.61E-07 | | [developmental growth](http://amigo.geneontology.org/amigo/term/GO:0048589) | 18 | 3.95 | 1.16E-02 | | [developmental process](http://amigo.geneontology.org/amigo/term/GO:0032502) | 106 | 1.86 | 2.80E-08 | | [growth](http://amigo.geneontology.org/amigo/term/GO:0040007) | 18 | 3.85 | 1.67E-02 | | [tube morphogenesis](http://amigo.geneontology.org/amigo/term/GO:0035239) | 29 | 3.83 | 1.05E-05 | | [anatomical structure morphogenesis](http://amigo.geneontology.org/amigo/term/GO:0009653) | 64 | 2.76 | 4.53E-10 | | [anatomical structure development](http://amigo.geneontology.org/amigo/term/GO:0048856) | 101 | 1.90 | 3.94E-08 | | [tube development](http://amigo.geneontology.org/amigo/term/GO:0035295) | 31 | 3.16 | 2.20E-04 | | [multicellular organism development](http://amigo.geneontology.org/amigo/term/GO:0007275) | 97 | 1.97 | 1.69E-08 | | [cell projection morphogenesis](http://amigo.geneontology.org/amigo/term/GO:0048858) | 19 | 3.73 | 1.35E-02 | | [cell part morphogenesis](http://amigo.geneontology.org/amigo/term/GO:0032990) | 28 | 3.66 | 5.63E-05 | | [cell part morphogenesis](http://amigo.geneontology.org/amigo/term/GO:0032989) | 19 | 3.55 | 2.70E-02 | | [cellular developmental process](http://amigo.geneontology.org/amigo/term/GO:0048869) | 68 | 1.83 | 3.09E-03 | | [regulation of system process](http://amigo.geneontology.org/amigo/term/GO:0044057) | 23 | 3.67 | 1.32E-03 | | [negative regulation of cell population proliferation](http://amigo.geneontology.org/amigo/term/GO:0008285) | 26 | 3.59 | 2.87E-04 | | [negative regulation of multicellular organismal process](http://amigo.geneontology.org/amigo/term/GO:0051241) | 40 | 3.58 | 3.75E-08 | | [negative regulation of developmental process](http://amigo.geneontology.org/amigo/term/GO:0051093) | 35 | 3.58 | 1.01E-06 | | [blood vessel development](http://amigo.geneontology.org/amigo/term/GO:0001568) | 19 | 3.42 | 4.44E-02 | | [vasculature development](http://amigo.geneontology.org/amigo/term/GO:0001944) | 20 | 3.41 | 2.58E-02 | | [circulatory system development](http://amigo.geneontology.org/amigo/term/GO:0072359) | 29 | 3.06 | 1.24E-03 | | [system development](http://amigo.geneontology.org/amigo/term/GO:0048731) | 85 | 1.98 | 6.77E-07 | | [positive regulation of apoptotic process](http://amigo.geneontology.org/amigo/term/GO:0043065) | 20 | 3.32 | 3.85E-02 | | [positive regulation of programmed cell death](http://amigo.geneontology.org/amigo/term/GO:0043068) | 21 | 3.39 | 1.58E-02 | | [positive regulation of cell death](http://amigo.geneontology.org/amigo/term/GO:0010942) | 23 | 3.33 | 6.77E-03 | | [positive regulation of cellular process](http://amigo.geneontology.org/amigo/term/GO:0048522) | 102 | 1.75 | 4.92E-06 | | positive regulation of cell motility | 20 | 3.31 | 3.95E-02 | | [positive regulation of cellular component movement](http://amigo.geneontology.org/amigo/term/GO:0051272) | 21 | 3.38 | 1.66E-02 | | [cell adhesion](http://amigo.geneontology.org/amigo/term/GO:0007155) | 27 | 3.11 | 2.59E-03 | | [biological adhesion](http://amigo.geneontology.org/amigo/term/GO:0022610) | 27 | 3.07 | 3.24E-03 | | [behavior](http://amigo.geneontology.org/amigo/term/GO:0007610) | 22 | 3.08 | 3.96E-02 | | [negative regulation of signal transduction](http://amigo.geneontology.org/amigo/term/GO:0009968) | 36 | 2.90 | 1.20E-04 | | [regulation of signal transduction](http://amigo.geneontology.org/amigo/term/GO:0009966) | 67 | 2.32 | 3.43E-07 | | [regulation of cell communication](http://amigo.geneontology.org/amigo/term/GO:0010646) | 73 | 2.21 | 2.16E-07 | | [regulation of signaling](http://amigo.geneontology.org/amigo/term/GO:0023051) | 72 | 2.17 | 7.96E-07 | | [negative regulation of cell communication](http://amigo.geneontology.org/amigo/term/GO:0010648) | 39 | 2.86 | 4.09E-05 | | [negative regulation of signaling](http://amigo.geneontology.org/amigo/term/GO:0023057) | 39 | 2.85 | 4.44E-05 | | [negative regulation of response to stimulus](http://amigo.geneontology.org/amigo/term/GO:0048585) | 49 | 2.99 | 6.00E-08 | | [anatomical structure formation involved in morphogenesis](http://amigo.geneontology.org/amigo/term/GO:0048646) | 28 | 2.88 | 6.78E-03 | | [tissue development](http://amigo.geneontology.org/amigo/term/GO:0009888) | 47 | 2.73 | 3.19E-06 | | [chemical homeostasis](http://amigo.geneontology.org/amigo/term/GO:0048878) | 31 | 2.66 | 8.72E-03 | | [regulation of biological quality](http://amigo.geneontology.org/amigo/term/GO:0065008) | 77 | 1.90 | 7.56E-05 | | [cellular response to organic substance](http://amigo.geneontology.org/amigo/term/GO:0071310) | 45 | 2.41 | 3.55E-04 | | [cellular response to chemical stimulus](http://amigo.geneontology.org/amigo/term/GO:0070887) | 57 | 2.33 | 1.17E-05 | | [response to chemical](http://amigo.geneontology.org/amigo/term/GO:0042221) | 77 | 2.16 | 2.20E-07 | | [response to organic substance](http://amigo.geneontology.org/amigo/term/GO:0010033) | 58 | 2.23 | 3.82E-05 | | [regulation of cell differentiation](http://amigo.geneontology.org/amigo/term/GO:0045595) | 39 | 2.40 | 4.00E-03 | | [regulation of molecular function](http://amigo.geneontology.org/amigo/term/GO:0065009) | 58 | 2.19 | 8.71E-05 | | response to external stimulus | 50 | 2.05 | 8.14E-03 | | animal organ development | 62 | 1.92 | 2.71E-03 | | [negative regulation of metabolic process](http://amigo.geneontology.org/amigo/term/GO:0009892) | 57 | 1.90 | 1.56E-02 | | regulation of metabolic process | 103 | 1.55 | 2.88E-03 | | [positive regulation of macromolecule metabolic process](http://amigo.geneontology.org/amigo/term/GO:0010604) | 63 | 1.86 | 7.18E-03 | | regulation of macromolecule metabolic process | 95 | 1.55 | 1.59E-02 | | [positive regulation of metabolic process](http://amigo.geneontology.org/amigo/term/GO:0009893) | 66 | 1.78 | 1.82E-02 | | [cell differentiation](http://amigo.geneontology.org/amigo/term/GO:0030154) | 67 | 1.83 | 4.17E-03 | | [localization](http://amigo.geneontology.org/amigo/term/GO:0051179) | 84 | 1.65 | 7.26E-03 | | regulation of primary metabolic process | 90 | 1.58 | 1.39E-02 | | | [**GO biological process**](http://pantherdb.org/tools/compareToRefList.jsp?sortOrder=1&sortList=categories)  *Mus musculus* (reference) | [Number](http://pantherdb.org/tools/compareToRefList.jsp?sortOrder=2&sortList=upload_1&sortField=num) of genes | [Fold Enrichment](http://pantherdb.org/tools/compareToRefList.jsp?sortOrder=2&sortList=upload_1&sortField=foldEnrich) | [P value](http://pantherdb.org/tools/compareToRefList.jsp?sortOrder=1&sortList=upload_1&sortField=pval) | | --- | --- | --- | --- | | protein hydroxylation | 5 | 39.49 | 3.17E-03 | | [response to chemical](http://amigo.geneontology.org/amigo/term/GO:0042221) | [34](http://pantherdb.org/tools/gxIdsList.do?acc=GO:0042221&list=upload_1&organism=Mus musculus) | 2.24 | 3.02E-02 | |  |  |  |  | |
|  |  |
| | [**GO molecular function**](http://pantherdb.org/tools/compareToRefList.jsp?sortOrder=1&sortList=categories) *Mus musculus* (reference) | [Number](http://pantherdb.org/tools/compareToRefList.jsp?sortOrder=2&sortList=upload_1&sortField=num) of genes | [Fold Enrichment](http://pantherdb.org/tools/compareToRefList.jsp?sortOrder=2&sortList=upload_1&sortField=foldEnrich) | [P value](http://pantherdb.org/tools/compareToRefList.jsp?sortOrder=1&sortList=upload_1&sortField=pval) | | --- | --- | --- | --- | | [collagen binding](http://amigo.geneontology.org/amigo/term/GO:0005518) | [7](http://pantherdb.org/tools/gxIdsList.do?acc=GO:0005518&list=upload_1&organism=Mus musculus) | 9.59 | 4.16E-02 | | [protein-containing complex binding](http://amigo.geneontology.org/amigo/term/GO:0044877) | [38](http://pantherdb.org/tools/gxIdsList.do?acc=GO:0044877&list=upload_1&organism=Mus musculus) | 2.50 | 9.19E-04 | | [binding](http://amigo.geneontology.org/amigo/term/GO:0005488) | [190](http://pantherdb.org/tools/gxIdsList.do?acc=GO:0005488&list=upload_1&organism=Mus musculus) | 1.35 | 3.30E-09 | | [extracellular matrix structural constituent](http://amigo.geneontology.org/amigo/term/GO:0005201) | [13](http://pantherdb.org/tools/gxIdsList.do?acc=GO:0005201&list=upload_1&organism=Mus musculus) | 8.43 | 3.68E-05 | | [glycosaminoglycan binding](http://amigo.geneontology.org/amigo/term/GO:0005539) | [12](http://pantherdb.org/tools/gxIdsList.do?acc=GO:0005539&list=upload_1&organism=Mus musculus) | 5.38 | 1.21E-02 | | [cell adhesion molecule binding](http://amigo.geneontology.org/amigo/term/GO:0050839) | [15](http://pantherdb.org/tools/gxIdsList.do?acc=GO:0050839&list=upload_1&organism=Mus musculus) | 5.08 | 1.49E-03 | | [protein binding](http://amigo.geneontology.org/amigo/term/GO:0005515) | [149](http://pantherdb.org/tools/gxIdsList.do?acc=GO:0005515&list=upload_1&organism=Mus musculus) | 1.57 | 2.24E-09 | | [carbohydrate binding](http://amigo.geneontology.org/amigo/term/GO:0030246) | [14](http://pantherdb.org/tools/gxIdsList.do?acc=GO:0030246&list=upload_1&organism=Mus musculus) | 4.97 | 4.63E-03 | | identical protein binding | [44](http://pantherdb.org/tools/gxIdsList.do?acc=GO:0042802&list=upload_1&organism=Mus musculus) | 1.96 | 4.86E-02 | | [metal ion binding](http://amigo.geneontology.org/amigo/term/GO:0046872) | [66](http://pantherdb.org/tools/gxIdsList.do?acc=GO:0046872&list=upload_1&organism=Mus musculus) | 1.82 | 2.94E-03 | | [cation binding](http://amigo.geneontology.org/amigo/term/GO:0043169) | [69](http://pantherdb.org/tools/gxIdsList.do?acc=GO:0043169&list=upload_1&organism=Mus musculus) | 1.85 | 5.62E-04 | | [ion binding](http://amigo.geneontology.org/amigo/term/GO:0043167) | [84](http://pantherdb.org/tools/gxIdsList.do?acc=GO:0043167&list=upload_1&organism=Mus musculus) | 1.56 | 2.67E-02 | | | [**GO molecular function**](http://pantherdb.org/tools/compareToRefList.jsp?sortOrder=1&sortList=categories) *Mus musculus* (reference) | [Number](http://pantherdb.org/tools/compareToRefList.jsp?sortOrder=2&sortList=upload_1&sortField=num) of genes | [Fold Enrichment](http://pantherdb.org/tools/compareToRefList.jsp?sortOrder=2&sortList=upload_1&sortField=foldEnrich) | [P value](http://pantherdb.org/tools/compareToRefList.jsp?sortOrder=1&sortList=upload_1&sortField=pval) | | --- | --- | --- | --- | | [inorganic molecular entity transmembrane transporter activity](http://amigo.geneontology.org/amigo/term/GO:0015318) | 15 | 3.52 | 7.05E-03 | | [transmembrane transporter activity](http://amigo.geneontology.org/amigo/term/GO:0022857) | 17 | 4.44 | 5.86E-03 | | [transporter activity](http://amigo.geneontology.org/amigo/term/GO:0005215) | 18 | 4.87 | 4.63E-03 | | oxidoreductase activity | 14 | 3.47 | 2.88E-02 | | ion transmembrane transporter activity | 16 | 3.96 | 6.27E-03 | |
|  |  |
| | [**GO cellular component**](http://pantherdb.org/tools/compareToRefList.jsp?sortOrder=1&sortList=categories)  *Mus musculus* (reference) | [Number](http://pantherdb.org/tools/compareToRefList.jsp?sortOrder=2&sortList=upload_1&sortField=num) of genes | [Fold Enrichment](http://pantherdb.org/tools/compareToRefList.jsp?sortOrder=2&sortList=upload_1&sortField=foldEnrich) | [P value](http://pantherdb.org/tools/compareToRefList.jsp?sortOrder=1&sortList=upload_1&sortField=pval) | | --- | --- | --- | --- | | [basement membrane](http://amigo.geneontology.org/amigo/term/GO:0005604) | [12](http://pantherdb.org/tools/gxIdsList.do?acc=GO:0005604&list=upload_1&organism=Mus musculus) | 10.06 | 1.12E-05 | | [collagen-containing extracellular matrix](http://amigo.geneontology.org/amigo/term/GO:0062023) | [28](http://pantherdb.org/tools/gxIdsList.do?acc=GO:0062023&list=upload_1&organism=Mus musculus) | 7.06 | 3.54E-12 | | [extracellular matrix](http://amigo.geneontology.org/amigo/term/GO:0031012) | [34](http://pantherdb.org/tools/gxIdsList.do?acc=GO:0031012&list=upload_1&organism=Mus musculus) | 6.40 | 4.54E-14 | | external encapsulating structure | [34](http://pantherdb.org/tools/gxIdsList.do?acc=GO:0030312&list=upload_1&organism=Mus musculus) | 6.37 | 5.07E-14 | | [extracellular region](http://amigo.geneontology.org/amigo/term/GO:0005576) | [119](http://pantherdb.org/tools/gxIdsList.do?acc=GO:0071944&list=upload_1&organism=Mus musculus) | 1.97 | 5.09E-13 | | [cellular anatomical entity](http://amigo.geneontology.org/amigo/term/GO:0110165) | [222](http://pantherdb.org/tools/gxIdsList.do?acc=GO:0110165&list=upload_1&organism=Mus musculus) | 1.14 | 3.20E-07 | | [basolateral plasma membrane](http://amigo.geneontology.org/amigo/term/GO:0016323) | [14](http://pantherdb.org/tools/gxIdsList.do?acc=GO:0016323&list=upload_1&organism=Mus musculus) | 5.14 | 1.64E-03 | | [plasma membrane region](http://amigo.geneontology.org/amigo/term/GO:0098590) | [33](http://pantherdb.org/tools/gxIdsList.do?acc=GO:0098590&list=upload_1&organism=Mus musculus) | 2.54 | 1.57E-03 | | [plasma membrane](http://amigo.geneontology.org/amigo/term/GO:0005886) | [95](http://pantherdb.org/tools/gxIdsList.do?acc=GO:0005886&list=upload_1&organism=Mus musculus) | 1.71 | 1.40E-05 | | [membrane](http://amigo.geneontology.org/amigo/term/GO:0016020) | [133](http://pantherdb.org/tools/gxIdsList.do?acc=GO:0016020&list=upload_1&organism=Mus musculus) | 1.36 | 5.56E-03 | | [plasma membrane protein complex](http://amigo.geneontology.org/amigo/term/GO:0098797) | [21](http://pantherdb.org/tools/gxIdsList.do?acc=GO:0098797&list=upload_1&organism=Mus musculus) | 3.68 | 7.27E-04 | | apical part of cell | [16](http://pantherdb.org/tools/gxIdsList.do?acc=GO:0045177&list=upload_1&organism=Mus musculus) | 3.39 | 4.57E-02 | | perinuclear region of cytoplasm | [20](http://pantherdb.org/tools/gxIdsList.do?acc=GO:0048471&list=upload_1&organism=Mus musculus) | 2.90 | 4.26E-02 | | [cytoplasm](http://amigo.geneontology.org/amigo/term/GO:0005737) | [155](http://pantherdb.org/tools/gxIdsList.do?acc=GO:0005737&list=upload_1&organism=Mus musculus) | 1.35 | 1.41E-04 | | [extracellular space](http://amigo.geneontology.org/amigo/term/GO:0005615) | [46](http://pantherdb.org/tools/gxIdsList.do?acc=GO:0005615&list=upload_1&organism=Mus musculus) | 2.17 | 1.32E-03 | | extracellular region | [66](http://pantherdb.org/tools/gxIdsList.do?acc=GO:0005576&list=upload_1&organism=Mus musculus) | 2.30 | 1.13E-07 | | [intrinsic component of plasma membrane](http://amigo.geneontology.org/amigo/term/GO:0031226) | [36](http://pantherdb.org/tools/gxIdsList.do?acc=GO:0031226&list=upload_1&organism=Mus musculus) | 2.16 | 2.49E-02 | | [cell junction](http://amigo.geneontology.org/amigo/term/GO:0030054) | [43](http://pantherdb.org/tools/gxIdsList.do?acc=GO:0030054&list=upload_1&organism=Mus musculus) | 2.03 | 1.37E-02 | | [endomembrane system](http://amigo.geneontology.org/amigo/term/GO:0012505) | [76](http://pantherdb.org/tools/gxIdsList.do?acc=GO:0012505&list=upload_1&organism=Mus musculus) | 1.86 | 4.07E-05 | | | [**GO cellular component**](http://pantherdb.org/tools/compareToRefList.jsp?sortOrder=1&sortList=categories)  *Mus musculus* (reference) | [Number](http://pantherdb.org/tools/compareToRefList.jsp?sortOrder=2&sortList=upload_1&sortField=num) of genes | [Fold Enrichment](http://pantherdb.org/tools/compareToRefList.jsp?sortOrder=2&sortList=upload_1&sortField=foldEnrich) | [P value](http://pantherdb.org/tools/compareToRefList.jsp?sortOrder=1&sortList=upload_1&sortField=pval) | | | --- | --- | --- | --- | --- | | collagen-containing extracellular matrix | [10](http://pantherdb.org/tools/gxIdsList.do?acc=GO:0062023&list=upload_1&organism=Mus musculus) | 5.93 | 1.28E-02 | | | extracellular matrix | [13](http://pantherdb.org/tools/gxIdsList.do?acc=GO:0031012&list=upload_1&organism=Mus musculus) | 5.76 | 7.23E-04 | | | external encapsulating structure | [13](http://pantherdb.org/tools/gxIdsList.do?acc=GO:0030312&list=upload_1&organism=Mus musculus) | 5.74 | 7.55E-04 | | | cell periphery | [51](http://pantherdb.org/tools/gxIdsList.do?acc=GO:0071944&list=upload_1&organism=Mus musculus) | 1.99 | 7.12E-05 | | | cellular anatomical entity | [95](http://pantherdb.org/tools/gxIdsList.do?acc=GO:0110165&list=upload_1&organism=Mus musculus) | 1.15 | 2.86E-02 | | | apical part of cell | [11](http://pantherdb.org/tools/gxIdsList.do?acc=GO:0045177&list=upload_1&organism=Mus musculus) | 5.49 | 9.12E-03 | | | extracellular region | [28](http://pantherdb.org/tools/gxIdsList.do?acc=GO:0005576&list=upload_1&organism=Mus musculus) | 2.29 | 2.73E-02 | | |  |  |  |  |  | |

**Supplementary Table S9: Affected biological processes. molecular functions and components from downregulated genes in midbrain and cortical NSCs cultivated for 13 days in physioxia.**

| **Midbrain NSCs** | **Cortex NSCs** |
| --- | --- |
| | [**GO biological process**](http://pantherdb.org/tools/compareToRefList.jsp?sortOrder=1&sortList=categories)  Mus musculus (REF) | [Number](http://pantherdb.org/tools/compareToRefList.jsp?sortOrder=2&sortList=upload_1&sortField=num) of genes | [Fold Enrichment](http://pantherdb.org/tools/compareToRefList.jsp?sortOrder=2&sortList=upload_1&sortField=foldEnrich) | [P value](http://pantherdb.org/tools/compareToRefList.jsp?sortOrder=1&sortList=upload_1&sortField=pval) | | --- | --- | --- | --- | | [regulation of Notch signaling pathway](http://amigo.geneontology.org/amigo/term/GO:0008593) | [7](http://pantherdb.org/tools/gxIdsList.do?acc=GO:0008593&list=upload_1&organism=Mus musculus) | 15.62 | 4.90E-03 | | [regulation of cell communication](http://amigo.geneontology.org/amigo/term/GO:0010646) | [37](http://pantherdb.org/tools/gxIdsList.do?acc=GO:0010646&list=upload_1&organism=Mus musculus) | 2.26 | 1.07E-02 | | [regulation of signaling](http://amigo.geneontology.org/amigo/term/GO:0023051) | [37](http://pantherdb.org/tools/gxIdsList.do?acc=GO:0023051&list=upload_1&organism=Mus musculus) | 2.25 | 1.13E-02 | | [positive regulation of neurogenesis](http://amigo.geneontology.org/amigo/term/GO:0050769) | [11](http://pantherdb.org/tools/gxIdsList.do?acc=GO:0050769&list=upload_1&organism=Mus musculus) | 7.60 | 2.85E-03 | | [positive regulation of nervous system development](http://amigo.geneontology.org/amigo/term/GO:0051962) | [13](http://pantherdb.org/tools/gxIdsList.do?acc=GO:0051962&list=upload_1&organism=Mus musculus) | 7.40 | 3.08E-04 | | [regulation of developmental process](http://amigo.geneontology.org/amigo/term/GO:0050793) | [31](http://pantherdb.org/tools/gxIdsList.do?acc=GO:0050793&list=upload_1&organism=Mus musculus) | 2.41 | 3.08E-02 | | [positive regulation of multicellular organismal process](http://amigo.geneontology.org/amigo/term/GO:0051240) | [24](http://pantherdb.org/tools/gxIdsList.do?acc=GO:0051240&list=upload_1&organism=Mus musculus) | 3.01 | 9.96E-03 | | [regulation of multicellular organismal process](http://amigo.geneontology.org/amigo/term/GO:0051239) | [33](http://pantherdb.org/tools/gxIdsList.do?acc=GO:0051239&list=upload_1&organism=Mus musculus) | 2.32 | 2.27E-02 | | [regulation of nervous system development](http://amigo.geneontology.org/amigo/term/GO:0051960) | [16](http://pantherdb.org/tools/gxIdsList.do?acc=GO:0051960&list=upload_1&organism=Mus musculus) | 5.88 | 1.65E-04 | | positive regulation of cell development | [12](http://pantherdb.org/tools/gxIdsList.do?acc=GO:0010720&list=upload_1&organism=Mus musculus) | 6.53 | 3.92E-03 | | positive regulation of cell differentiation | [17](http://pantherdb.org/tools/gxIdsList.do?acc=GO:0045597&list=upload_1&organism=Mus musculus) | 3.60 | 4.83E-02 | | [regulation of cell development](http://amigo.geneontology.org/amigo/term/GO:0010720) | [16](http://pantherdb.org/tools/gxIdsList.do?acc=GO:0060284&list=upload_1&organism=Mus musculus) | 5.45 | 4.57E-04 | | [regulation of neurogenesis](http://amigo.geneontology.org/amigo/term/GO:0050767) | [14](http://pantherdb.org/tools/gxIdsList.do?acc=GO:0050767&list=upload_1&organism=Mus musculus) | 6.30 | 6.06E-04 | | [generation of neurons](http://amigo.geneontology.org/amigo/term/GO:0048699) | [23](http://pantherdb.org/tools/gxIdsList.do?acc=GO:0048699&list=upload_1&organism=Mus musculus) | 3.31 | 3.44E-03 | | [neurogenesis](http://amigo.geneontology.org/amigo/term/GO:0022008) | [24](http://pantherdb.org/tools/gxIdsList.do?acc=GO:0022008&list=upload_1&organism=Mus musculus) | 3.15 | 4.40E-03 | | [cell differentiation](http://amigo.geneontology.org/amigo/term/GO:0030154) | [39](http://pantherdb.org/tools/gxIdsList.do?acc=GO:0030154&list=upload_1&organism=Mus musculus) | 2.15 | 1.67E-02 | | [cellular developmental process](http://amigo.geneontology.org/amigo/term/GO:0048869) | [39](http://pantherdb.org/tools/gxIdsList.do?acc=GO:0048869&list=upload_1&organism=Mus musculus) | 2.12 | 2.05E-02 | | [nervous system development](http://amigo.geneontology.org/amigo/term/GO:0007399) | [29](http://pantherdb.org/tools/gxIdsList.do?acc=GO:0007399&list=upload_1&organism=Mus musculus) | 2.72 | 4.80E-03 | | [system development](http://amigo.geneontology.org/amigo/term/GO:0048731) | [44](http://pantherdb.org/tools/gxIdsList.do?acc=GO:0048731&list=upload_1&organism=Mus musculus) | 2.07 | 6.19E-03 | | [multicellular organism development](http://amigo.geneontology.org/amigo/term/GO:0007275) | [46](http://pantherdb.org/tools/gxIdsList.do?acc=GO:0007275&list=upload_1&organism=Mus musculus) | 1.89 | 4.46E-02 | | [cell-cell signaling](http://amigo.geneontology.org/amigo/term/GO:0007267) | [19](http://pantherdb.org/tools/gxIdsList.do?acc=GO:0007267&list=upload_1&organism=Mus musculus) | 4.54 | 3.85E-04 | | [regulation of plasma membrane bounded cell projection organization](http://amigo.geneontology.org/amigo/term/GO:0120035) | [16](http://pantherdb.org/tools/gxIdsList.do?acc=GO:0120035&list=upload_1&organism=Mus musculus) | 4.30 | 1.01E-02 | | [regulation of cell projection organization](http://amigo.geneontology.org/amigo/term/GO:0031344) | [16](http://pantherdb.org/tools/gxIdsList.do?acc=GO:0031344&list=upload_1&organism=Mus musculus) | 4.21 | 1.34E-02 | | [behavior](http://amigo.geneontology.org/amigo/term/GO:0007610) | [15](http://pantherdb.org/tools/gxIdsList.do?acc=GO:0007610&list=upload_1&organism=Mus musculus) | 4.24 | 2.68E-02 | | [regulation of anatomical structure morphogenesis](http://amigo.geneontology.org/amigo/term/GO:0022603) | [18](http://pantherdb.org/tools/gxIdsList.do?acc=GO:0022603&list=upload_1&organism=Mus musculus) | 3.65 | 2.04E-02 | | | [**GO biological process**](http://pantherdb.org/tools/compareToRefList.jsp?sortOrder=1&sortList=categories)  Mus musculus (REF) | [Number](http://pantherdb.org/tools/compareToRefList.jsp?sortOrder=2&sortList=upload_1&sortField=num) of genes | [Fold Enrichment](http://pantherdb.org/tools/compareToRefList.jsp?sortOrder=2&sortList=upload_1&sortField=foldEnrich) | [P value](http://pantherdb.org/tools/compareToRefList.jsp?sortOrder=1&sortList=upload_1&sortField=pval) | | | --- | --- | --- | --- | --- | | None | | | |  | |
|  |  |
| | **GO molecular function**  [Mus musculus](http://pantherdb.org/tools/gxIdsList.do?reflist=1) (REF) | [Number](http://pantherdb.org/tools/compareToRefList.jsp?sortOrder=2&sortList=upload_1&sortField=num) of genes | [Fold Enrichment](http://pantherdb.org/tools/compareToRefList.jsp?sortOrder=2&sortList=upload_1&sortField=foldEnrich) | [P value](http://pantherdb.org/tools/compareToRefList.jsp?sortOrder=1&sortList=upload_1&sortField=pval) | | --- | --- | --- | --- | | [phosphoric diester hydrolase activity](http://amigo.geneontology.org/amigo/term/GO:0008081) | [6](http://pantherdb.org/tools/gxIdsList.do?acc=GO:0005518&list=upload_1&organism=Mus musculus) | 14.19 | 1.73E-02 | | | [**GO molecular function**](http://pantherdb.org/tools/compareToRefList.jsp?sortOrder=1&sortList=categories)  Mus musculus (REF) | [Number](http://pantherdb.org/tools/compareToRefList.jsp?sortOrder=2&sortList=upload_1&sortField=num) of genes | [Fold Enrichment](http://pantherdb.org/tools/compareToRefList.jsp?sortOrder=2&sortList=upload_1&sortField=foldEnrich) | [P value](http://pantherdb.org/tools/compareToRefList.jsp?sortOrder=1&sortList=upload_1&sortField=pval) | | --- | --- | --- | --- | | cyclic-nucleotide-mediated signaling | [15](http://pantherdb.org/tools/gxIdsList.do?acc=GO:0015075&list=upload_1&organism=Mus musculus) | 26.02 | 1.23E-02 | | adenylate cyclase-modulating G protein-coupled receptor signaling pathway | 5 | 21.56 | 3.02E-02 | | regulation of biological quality | 5 | 3.35 | 2.99E-02 | |
|  |  |
| | [**GO cellular component**](http://pantherdb.org/tools/compareToRefList.jsp?sortOrder=1&sortList=categories)  Mus musculus (REF) | [Number](http://pantherdb.org/tools/compareToRefList.jsp?sortOrder=2&sortList=upload_1&sortField=num) of genes | [Fold Enrichment](http://pantherdb.org/tools/compareToRefList.jsp?sortOrder=2&sortList=upload_1&sortField=foldEnrich) | [P value](http://pantherdb.org/tools/compareToRefList.jsp?sortOrder=1&sortList=upload_1&sortField=pval) | | --- | --- | --- | --- | | Schaffer collateral - CA1 synapse | [6](http://pantherdb.org/tools/gxIdsList.do?acc=GO:0098685&list=upload_1&organism=Mus musculus) | 10.42 | 4.70E-02 | | cell junction | [26](http://pantherdb.org/tools/gxIdsList.do?acc=GO:0030054&list=upload_1&organism=Mus musculus) | 2.48 | 1.78E-02 | | [integral component of postsynaptic membrane](http://amigo.geneontology.org/amigo/term/GO:0099055) | [7](http://pantherdb.org/tools/gxIdsList.do?acc=GO:0099055&list=upload_1&organism=Mus musculus) | 8.13 | 4.72E-02 | | [integral component of synaptic membrane](http://amigo.geneontology.org/amigo/term/GO:0099699) | [8](http://pantherdb.org/tools/gxIdsList.do?acc=GO:0099699&list=upload_1&organism=Mus musculus) | 6.95 | 3.77E-02 | | [integral component of membrane](http://amigo.geneontology.org/amigo/term/GO:0099699) | [51](http://pantherdb.org/tools/gxIdsList.do?acc=GO:0016021&list=upload_1&organism=Mus musculus) | 1.70 | 3.42E-02 | | [intrinsic component of membrane](http://amigo.geneontology.org/amigo/term/GO:0031224) | [53](http://pantherdb.org/tools/gxIdsList.do?acc=GO:0031224&list=upload_1&organism=Mus musculus) | 1.72 | 1.51E-02 | | [membrane](http://amigo.geneontology.org/amigo/term/GO:0016020) | [75](http://pantherdb.org/tools/gxIdsList.do?acc=GO:0016020&list=upload_1&organism=Mus musculus) | 1.55 | 1.01E-03 | | [plasma membrane](http://amigo.geneontology.org/amigo/term/GO:0005886) | [54](http://pantherdb.org/tools/gxIdsList.do?acc=GO:0005886&list=upload_1&organism=Mus musculus) | 1.97 | 9.22E-05 | | [cell periphery](http://amigo.geneontology.org/amigo/term/GO:0071944) | [55](http://pantherdb.org/tools/gxIdsList.do?acc=GO:0071944&list=upload_1&organism=Mus musculus) | 1.84 | 7.61E-04 | | [postsynapse](http://amigo.geneontology.org/amigo/term/GO:0098794) | [14](http://pantherdb.org/tools/gxIdsList.do?acc=GO:0098794&list=upload_1&organism=Mus musculus) | 3.74 | 3.74E-02 | | [postsynaptic density](http://amigo.geneontology.org/amigo/term/GO:0014069) | [11](http://pantherdb.org/tools/gxIdsList.do?acc=GO:0014069&list=upload_1&organism=Mus musculus) | 5.44 | 1.07E-02 | | [asymmetric synapse](http://amigo.geneontology.org/amigo/term/GO:0032279) | [11](http://pantherdb.org/tools/gxIdsList.do?acc=GO:0032279&list=upload_1&organism=Mus musculus) | 5.37 | 1.20E-02 | | [neuron to neuron synapse](http://amigo.geneontology.org/amigo/term/GO:0098984) | [11](http://pantherdb.org/tools/gxIdsList.do?acc=GO:0098984&list=upload_1&organism=Mus musculus) | 5.02 | 2.21E-02 | | [postsynaptic specialization](http://amigo.geneontology.org/amigo/term/GO:0099572) | [11](http://pantherdb.org/tools/gxIdsList.do?acc=GO:0099572&list=upload_1&organism=Mus musculus) | 4.95 | 2.51E-02 | | | [**GO cellular component**](http://pantherdb.org/tools/compareToRefList.jsp?sortOrder=1&sortList=categories)  Mus musculus (REF) | [Number](http://pantherdb.org/tools/compareToRefList.jsp?sortOrder=2&sortList=upload_1&sortField=num) of genes | [Fold Enrichment](http://pantherdb.org/tools/compareToRefList.jsp?sortOrder=2&sortList=upload_1&sortField=foldEnrich) | [P value](http://pantherdb.org/tools/compareToRefList.jsp?sortOrder=1&sortList=upload_1&sortField=pval) | | | --- | --- | --- | --- | --- | | [cytoplasm](http://amigo.geneontology.org/amigo/term/GO:0005737) | [23](http://pantherdb.org/tools/gxIdsList.do?acc=GO:0005737&list=upload_1&organism=Mus musculus) | 1.81 | 3.01E-02 | | |  |  |  |  |  | |
